# Emotion Regulation Using Virtual Environments and Real-Time fMRI Neurofeedback

**DOI:** 10.3389/fneur.2018.00390

**Published:** 2018-07-24

**Authors:** Valentina Lorenzetti, Bruno Melo, Rodrigo Basílio, Chao Suo, Murat Yücel, Carlos J. Tierra-Criollo, Jorge Moll

**Affiliations:** ^1^School of Psychology, Faculty of Health Sciences, Australian Catholic University, Melbourne, VIC, Australia; ^2^Department of Psychological Sciences, Institute of Psychology Health and Society, University of Liverpool, Liverpool, United Kingdom; ^3^Brain and Mental Health Laboratory, School of Psychological Sciences and Monash Institute of Cognitive and Clinical Neurosciences, Monash University, Melbourne, VIC, Australia; ^4^D'Or Institute for Research and Education, IDOR, Rio de Janeiro, Brazil; ^5^Biomedical Engineering Program, COPPE, Federal University of Rio de Janeiro, Rio de Janeiro, Brazil

**Keywords:** fMRI, emotion regulation, neurofeedback, BCI, region of interest, support vector machine, virtual reality, virtual environments

## Abstract

Neurofeedback (NFB) enables the voluntary regulation of brain activity, with promising applications to enhance and recover emotion and cognitive processes, and their underlying neurobiology. It remains unclear whether NFB can be used to aid and sustain complex emotions, with ecological validity implications. We provide a technical proof of concept of a novel real-time functional magnetic resonance imaging (rtfMRI) NFB procedure. Using rtfMRI-NFB, we enabled participants to voluntarily enhance their own neural activity while they experienced complex emotions. The rtfMRI-NFB software (FRIEND Engine) was adapted to provide a virtual environment as brain computer interface (BCI) and musical excerpts to induce two emotions (tenderness and anguish), aided by participants' preferred personalized strategies to maximize the intensity of these emotions. Eight participants from two experimental sites performed rtfMRI-NFB on two consecutive days in a counterbalanced design. On one day, rtfMRI-NFB was delivered to participants using a region of interest (ROI) method, while on the other day using a support vector machine (SVM) classifier. Our multimodal VR/NFB approach was technically feasible and robust as a method for real-time measurement of the neural correlates of complex emotional states and their voluntary modulation. Guided by the color changes of the virtual environment BCI during rtfMRI-NFB, participants successfully increased in real time, the activity of the septo-hypothalamic area and the amygdala during the ROI based rtfMRI-NFB, and successfully evoked distributed patterns of brain activity classified as tenderness and anguish during SVM-based rtfMRI-NFB. Offline fMRI analyses confirmed that during tenderness rtfMRI-NFB conditions, participants recruited the septo-hypothalamic area and other regions ascribed to social affiliative emotions (medial frontal / temporal pole and precuneus). During anguish rtfMRI-NFB conditions, participants recruited the amygdala and other dorsolateral prefrontal and additional regions associated with negative affect. These findings were robust and were demonstrable at the individual subject level, and were reflected in self-reported emotion intensity during rtfMRI-NFB, being observed with both ROI and SVM methods and across the two sites. Our multimodal VR/rtfMRI-NFB protocol provides an engaging tool for brain-based interventions to enhance emotional states in healthy subjects and may find applications in clinical conditions associated with anxiety, stress and impaired empathy among others.

## Introduction

Neurofeedback (NFB) is a novel application of brain-computer interfaces that aids real-time voluntarily regulation of brain activity. Mounting evidence shows that NFB has promising effects to enhance behavior, cognitive and emotional processes in normative samples (1–5). NFB has also been preliminary used to restore aberrant neurobiology and symptoms in neurological conditions (e.g., stroke, traumatic brain injury) and in psychopathology (e.g., ADHD, autism, depression, addiction) (1–7). Real-time functional magnetic resonance imaging (rtfMRI) based NFB has the potential to provide insight in understanding the mechanisms of psychological states (8–10). These include affiliative emotions (11) underpinned by deep brain nuclei (12, 13) the activity of which is unlikely to be robustly measured via surface electroencephalography.

rtfMRI NFB tools can be used to study the causal mechanisms of complex emotions and to inform evidence-based personalized interventions to enhance and recover aberrant emotional states (and their neural substrates) in normative and clinical samples. One key practical human challenge of fMRI studies includes participants being distracted and experiencing difficulties to feel valid psychological states in the scanner environment, particularly when trying to sustain complex emotions.

Recent studies have combined immersive virtual environments with multiple sensory modalities to deliver psychological/cognitive interventions, and to enhance their effectiveness via engaging and motivating individuals to practice (14–16).

Only two proof of concept studies have combined rt-NFB with virtual environments as brain computer interfaces (BCI). An electroencephalography-based NFB study computed brain activity from about 500 participants collectively, during an interactive game of relaxation and concentration over one night (16), where individual's level of brain activity could not be discerned. A separate rtfMRI-NFB paradigm used a virtual fire interface to up-regulate and down-regulate brain activity in eight healthy participants—but this was devoid of any emotional states and far from multimodal and immersive (17).

It remains untested whether rt-NFB platforms integrating multisensory virtual environments can successfully recruit complex emotions and sustain these emotions long and strong enough to probe their underlying neural correlates. Such a platform can advance NFB applications, via (i) increasing the ecological validity of rtfMRI-NFB experiments, and their relevance for the daily experiences of emotions outside of experimental settings, (ii) adapting NFB interfaces to the individual and target population so these are more relatable, engaging and effective in generating and sustaining complex emotions that maximize the success of rtfMRI-NFB interventions (18–20).

This study aims to demonstrate the feasibility of an engaging rtfMRI-NFB interface that can be individually tailored and, specifically, to provide a proof of concept for a rtfMRI-NFB integrating a virtual environment as a BCI and musical stimuli using both local (region of interest, ROI) and distributed (support vector machine, SVM) analyses. The FRIEND Engine Framework system (21) was enhanced and adapted for this aim. We recruited healthy young adults performing rtfMRI-NFB during complex emotion experiences, including tenderness—a positive affiliative emotion - and anguish—a self-reflective negative emotion (11, 13, 22–25).

We also aimed to validate the functional anatomy of these complex emotions during rtfMRI-NFB. After the real-time data was collected, we ran offline fMRI data analyses to verify the effects of the real-time neurofeedback task on brain activity using standard preprocessing and statistical analysis methods.

We hypothesized that participants would voluntary change the color of a virtual environment in the BCI during rtfMRI-NFB using the activity of the following regions: (i) for the tenderness condition, the septo-hypothalamic area (when using ROI-based rtfMRI-NFB method) and other brain areas ascribed to positive affiliative emotions i.e., medial orbitofrontal areas (when using SVM-based rtfMRI-NFB method) (11, 25–27); and (ii) for the anguish condition, the amygdala (during the ROI-based fMRI-NFB method) and also lateral prefrontal cortices implicated in negative affect (e.g., anguish, fear, anxiety, negative mood, stress, psychological pain), and in psychopathologies where negative affect is a feature [e.g., depression and generalized anxiety disorder (28–32)] (during SVM-based rtfMRI-NFB).

## Materials and methods

### Participants

We used a single subject, repeated measures design with two identical assessments on two consecutive days, counterbalanced by rtfMRI-NFB method (i.e., ROI and SVM). We recruited eight psychiatrically and neurologically healthy postgraduate research students, free of psychoactive medication and with normal or corrected-to-normal vision. Four participants were recruited from the D'Or Institute for Research and Education (IDOR) in Rio de Janeiro, Brazil (approved by the Ethics and Scientific committees of the Copa D'Or Hospital, Rio de Janeiro, Brazil - No 922.218). To validate the protocol in a different scanner and institution, we also recruited four participants from the Monash Biomedical Imaging (MBI) at Monash University in Melbourne, Australia (MUHREC CF15/1756 - 2015000893). All volunteers provided written informed consent prior to study participation.

### Design of the neurofeedback BCI

**Figure 1:**
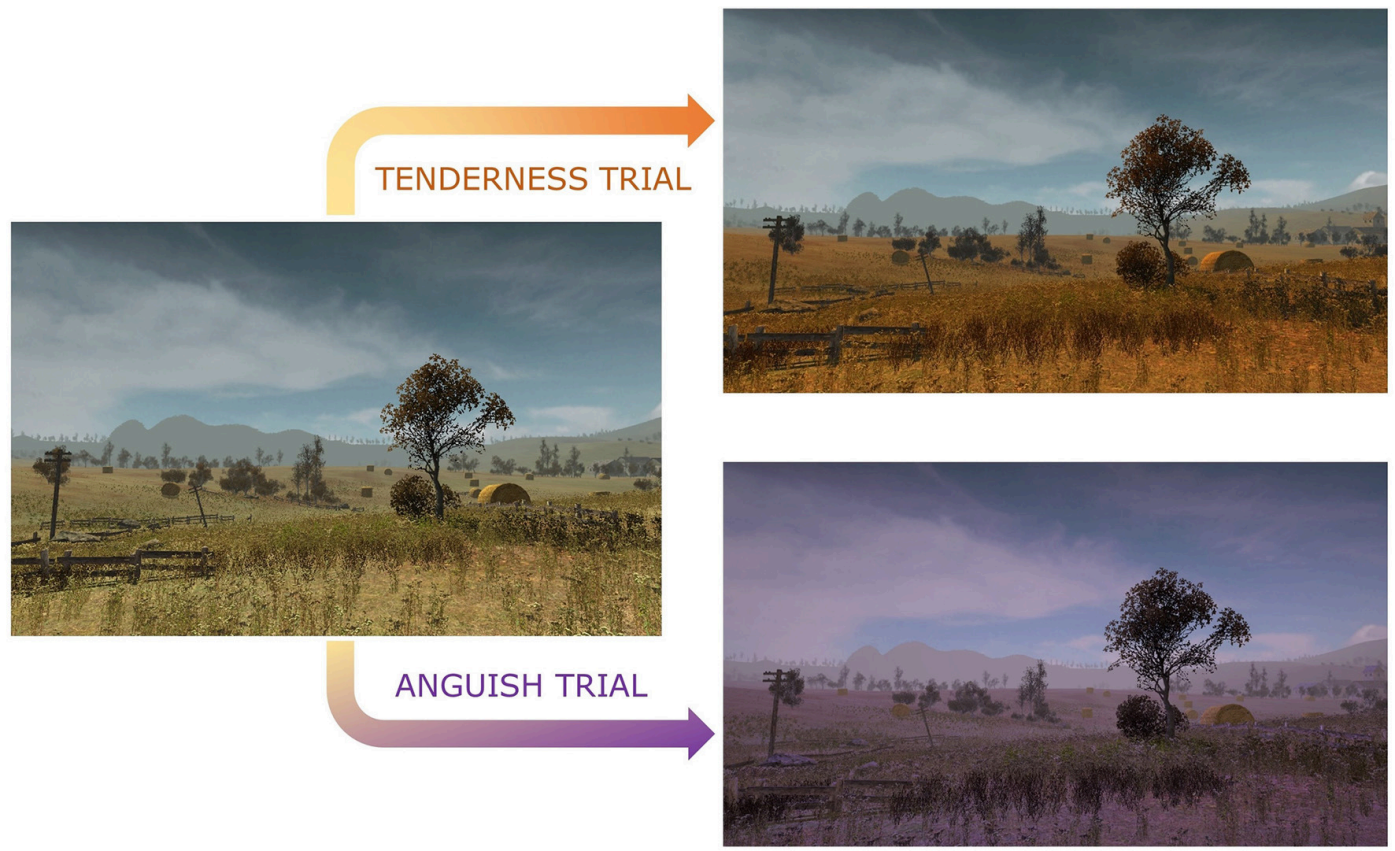
Color hue modulation of the virtual environment during rtfMRI-NFB. The color hue changes from baseline neutral trials to a more intense orange and purple as participants increasingly engage target brain regions for tenderness and anguish trials.

Supplementary video 1 and Figure 1 show the BCI used for the rt-fMRI NFB. The BCI comprised a virtual environment as a medium to convey sensory feedback to participants in real time, in association with ongoing tenderness, anguish and neutral emotional states. The virtual environment was created by editing the Unity 3D asset Autumnal Nature Pack (Unity 3D, https://assetstore.unity.com/packages/3d/environments/autumnal-nature-pack-3649) and displayed a first-person navigation at walking speed through hills and cornfields, with a total duration of 10′8″ (Supplementary Video 1). The virtual environment was prepared to alternate between different trial types: neutral (30″), tenderness (46″) and anguish (46″).

The trial types were displayed via changes in the base color hues of the virtual environment and via specific music excerpts. Music excerpts were fixed for each trial type, and not influenced by current neural/psychological states (no music for *Neutral*, mild, gentle music for *Tenderness* and eerie, distorted music for *Anguish*). Music excerpts were selected from 20 audio tracks, all normalized using the root mean square feature of Audacity software (Audacity, http://www.audacityteam.org). The audio tracks were previously rated to have comparable volume, pace, and rhythm. For the rtfMRI-NFB task runs, four excerpts for tenderness and four excerpts for anguish were played.

Neutral trials were characterized by a normal colored virtual landscape displayed in the BCI with no background music. Tenderness trials were characterized by a change in the color of the virtual landscape to orange and were accompanied by tenderness music excerpts. Anguish trials commenced when the color of the environment turned to purple hues and were accompanied by anguish music excerpts.

### Neurofeedback task

#### Task practice outside the MRI

For training purposes, we recorded a video showing a sample of the virtual environment. The video lasted as long as one run of the rtfMRI-NFB task (10′ 8″) and was used by participants to practice tenderness, anguish and neutral states before the MRI. With this practice, participants could learn which music tracks and VR color changes in the BCI corresponded to tenderness, anguish and neutral trials.

#### Neurofeedback interface

As shown in Figure 1, instead of a classic thermometer, the color of the virtual environment was used as BCI changed in real time with increased engagement of the neural activity/pattern corresponding to distinct target emotional states—orange for tenderness trials, purple for anguish trials and natural light tones for neutral trials. Participants were instructed to experience tenderness or anguish as intensely as possible in the respective trials and to increase the intensity of their emotion to turn in real time, the color of the virtual environment BCI to as orange as possible during tenderness trials, and as purple as possible during anguish trials, which increased in turn the corresponding neural activity/pattern.

#### Training run

During the training MRI run for rtfMRI-NFB, participants were instructed to feel the tenderness, anguish and neutral states as intensely as possible. This allowed mapping the brain regions that were most engaged by each individual while experiencing the emotions. We mapped and used the activity in these brain regions for each participant as a source for rtfMRI-NFB. The musical stimuli were delivered with MRI-compatible headphones (MR-Confon, http://www.mr-confon.de). The volume of the song excerpts was adjusted for each participant to a level where they could comfortably hear the music while performing rtfMRI-NFB.

#### Neurofeedback task

For half of the sample, the rtfMRI-NFB task started with a tenderness trials block at baseline and follow up. The other half started the task with an anguish trials block at both assessments.

The fMRI protocol comprised four runs: a training run and three rtfMRI-NFB runs (10′8″ each). The training run allowed mapping which brain regions the participant engaged while experiencing tenderness and anguish. The three subsequent rtfMRI-NFB runs provided participants with continuous feedback (every 2″) on their brain activity in the form of updating the color of the virtual scenario in the BCI. The more participants engaged the target brain regions corresponding to tenderness and anguish states, the more the virtual environment would turn into orange and purple shades, respectively. During neutral rtfMRI-NFB trials, participants were not required to change the color of the virtual environment and this remained at baseline color.

#### Neurofeedback methods: ROI and SVM

rtfMRI-NFB was delivered online and continuously via an updated platform of the FRIEND Engine Framework v 0.5 (21). We defined feedback signal as a sensory input to the participant (i.e., the color hue saturation of the dynamic virtual scenarios presented visually to participants in the BCI). This metric was determined by a number reflecting the hemodynamic state of *a priori* brain regions (or network of regions). Participants were instructed to enhance the target emotion as to intensify the color hue of the virtual environment BCI from neutral (baseline scenario hue) to orange (tenderness trials) or to purple (anguish trials).

**Figure 2:**
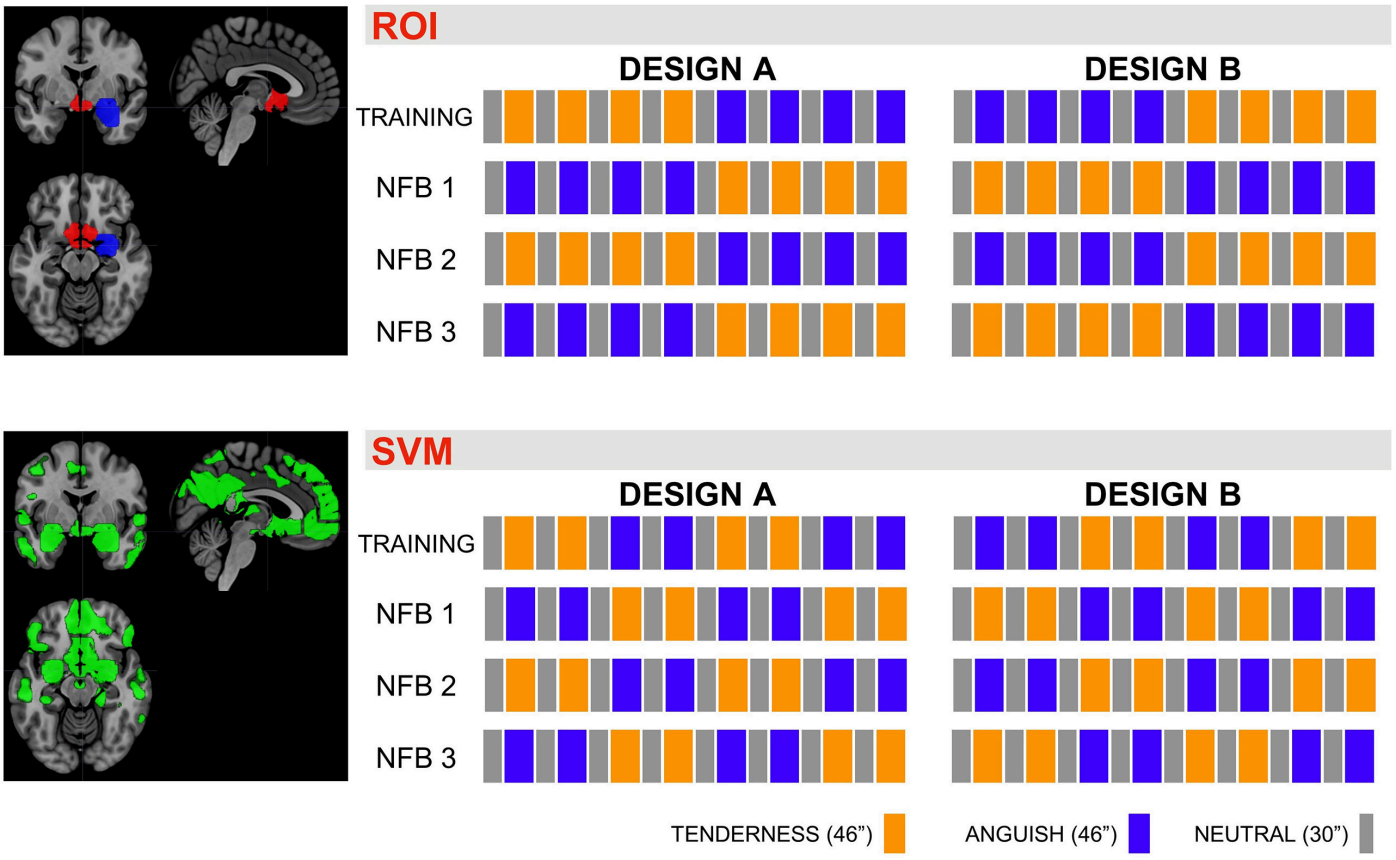
Design of the NFB trials. Presentation order for the emotion blocks Neutral (“N,” gray boxes, 30″ per block, with no music in the background), Tenderness (“T,” orange boxes, 46″ per block, while playing one of the four tenderness music tracks) and Anguish (“A,” blue boxes, 46″ per block, while playing one of the four anguish music tracks). The emotion blocks order was counterbalanced across trials A and B and runs 1–4. The sessions using the ROI = region of interest NFB method alternated the emotion blocks (top half) and the sessions using the SVM = support vector machine method presented the emotion trial blocks consecutively (bottom half), to reliably detect brain activity patterns.

We used two different rtfMRI-NFB methods to compute brain activity unknowingly to participants, test the capability of this software and explore whether the patterns of brain activity were more robustly recruited via either SVM based rtfMRI-NFB or ROI based rtfMRI-NFB. Half of the sample was randomly allocated to SVM method on day one and ROI method on day two, and the opposite order was used for the other half. We counterbalanced the presentation of the emotion trial types (Figure 2). The visual feedback on participants' brain activity was equivalent across ROI and SVM acquisitions although these relied on different metrics.

The rtfMRI-NFB ROI method computed percentage signal change (PSC) within the 10% most active voxels with an a priori defined ROI, measured across four blocks of the first training NFB run. ROIs included the septo-hypothalamic area when contrasting tenderness versus neutral conditions (11) and the right amygdala for when contrasting anguish versus neutral (33, 34). The feedback value was given by the equation

$$\begin{array}{r} \frac{{\bar{ROI}}_{curr\text{\_}vol}-\overset{B}{\underset{k=1}{\sum }}\frac{1}{sig\left(k\right)}\overset{B}{\underset{k=1}{\sum }}{sig\left(k\right)\;\bar{ROI}}_{k}}{\overset{B}{\underset{k=1}{\sum }}\frac{1}{sig\left(k\right)}\overset{B}{\underset{k=1}{\sum }}{sig\left(k\right)\;\bar{ROI}}_{k}}\;, \end{array}$$

where *ROI_curr_vol_* is the mean of the ROI on the current volume, *B* is the number of volumes in the previous baseline condition and *ROI_k_* is the mean of the *k*th volume (21) weighted by a sigmoid function, *sig*(*k*). The feedback value was used to modulate the color of the virtual environment, so that the higher the percent signal change, the more participants changed the color to orange and purple for the conditions of tenderness and anguish, respectively.

The SVM rtfMRI-NFB method provides the distance of a new observation relative to a separating hyperplane. It is a multivariate pattern analysis method that classifies the pattern of brain activity that best segregates between distinct conditions, which in our study comprised tenderness and anguish (i.e., all computed relative to the previous neutral block). We used a SVM classifier with a linear kernel and a cumulative training, meaning that all brain activity patterns observed during the rtfMRI-NFB task thus far are used at the end of the run to retrain and update the SVM classifier/model to use in the following runs. The projected value of a new observation was used to define the neurofeedback information, in our case, the color tonality of the virtual environment. For a new image volume, composed by real numbers *x*^*t*^, the projected value was given by *x*^*t*^*w*+*b*, where *w* is a vector containing the hyperplane coefficients and *b* is a constant (21). The more the pattern of brain activity segregated/classified the conditions, the more the color of the virtual landscape in the BCI turned to orange and purple, respectively.

The SVM rtfMRI-NFB method used a feature selection mask that included brain regions implicated in positive affiliative emotions (e.g., frontal, temporal, parietal and subcortical areas), and that excluded from SVM training and decoding those areas involved in sensorimotor or visuospatial processing (11).

### MRI data acquisition

MRI and rtfMRI-NFB data were acquired in the two sites using a 3T Philips Achieva - at the D'Or Institute for Research and Education, in Rio de Janeiro, Brazil (Site 1) - and a 3T Siemens Magnetom Skyra - at the Monash Biomedical Imaging facility and the Brain and Mental Health Imaging laboratory, Monash Institute of Cognitive and Clinical Neurosciences, Monash University in Melbourne, Australia (Site 2).

Immediately before the rtfMRI-NFB task, we acquired high-resolution anatomical images. In Site 1 we used an isotropic T1-weighted 3D turbo field echo sequence (TR/TE = 7.2/3.4 (s), flip angle = 8°, matrix size 240 × 240, FOV = 240 mm^2^, slice thickness = 1 mm, 170 slices, slice order ascending). Head motion was minimized via foam padding and straps over the forehead and under the chin. In Site 2 we used an isotropic T1 MP-RAGE scan (with TR/TE = 2.3/2.0 (s), flip angle = 9°, matrix size 256 × 240, FOV = 256 × 240 (mm), slice thickness = 1 mm, 170 slices, slice order descending).

fMRI data from the training run and the rtfMRI-NFB task comprised a total of 1,216 EPI volumes acquired over 40′32″ in four runs (i.e., each run comprised 304 volumes and lasted 10′8″). In both sites fMRI data were acquired with T2^*^-weighted EPI (BOLD contrast), with TR/TE = 2,000/22 (ms), matrix = 64 × 64, FOV = 240 mm^2^, flip angle = 90°, isotropic voxel = 3.75 mm^3^, 24 slices. Before each fMRI run, we collected five dummy volumes for T1 equilibration. In Site 1 we used an optimized sequence with SENSE factor of 1.5 and dynamic stabilization to enhance temporal signal-to-noise (35) in brain areas prone to susceptibility effects (i.e., basal forebrain, ventromedial prefrontal cortex).

### Behavioral methods

**Figure 3:**
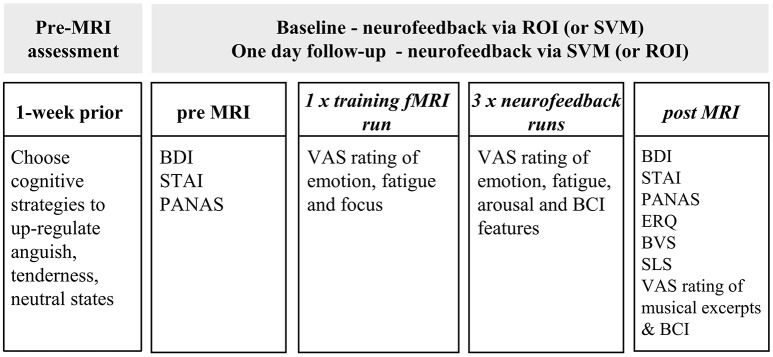
Outline of the assessment protocol. Assessments were identical across sites and days (baseline and day 2), with questionnaires administration before, during and after the MRI assessment and training/NFB runs. Unknowingly, each participant was delivered NFB using a distinct NFB method (either SVM = support vector machine or ROI = region of interest) on each of the two assessment days (gray box). Half of the participants delivered a ROI NFB method at baseline and a SVM NFB method at the one-day follow up. The other half underwent SVM NFB first and ROI NFB at follow up. BDI = Beck Depression Inventory (36), ROI = region of interest; STAI = Spielberger State and Trait Anxiety Inventory (37); PANAS = Positive and Negative Affect Scale (38). ERQ = Emotion Regulation Questionnaire (39), BVS = Body Vigilance Scale (40), SLS = Satisfaction with Life Scale (41).

The assessment protocol is overviewed in Figure 3. One week before baseline assessment, participants were contacted to identify the most effective personalized cognitive strategies to elicit tenderness, anguish and neutral states that would have been used by them during rtfMRI-NFB to up-regulate the underlying neural substrates. Tenderness was defined as a positive and affiliative (but not romantic) emotion experienced toward significant others, anguish as a negative and upsetting emotion not necessarily involving others, and neutral as emotionally neutral. Participants were also provided with a list of 20 sentences or mantras for each emotion, to use as a source to reflect on cognitive strategies to elicit tenderness and anguish states.

At baseline assessment, we collected participants socio-demographic data. At both baseline and 1 day follow up assessment, we administered questionnaires immediately before and after the MRI scan and rtfMRI-NFB to monitor changes in affect (Supplementary Table 1). Questionnaires included the *Beck Depression Inventory* [BDI (36)], the “state” subscale of the *State and Trait Anxiety Inventory* [STAI (37)] and *Positive And Negative Affect Scale* [PANAS (38)].

We administered visual analog scales (VAS) in between all the MRI runs (comprising a training run and three rtfMRI-NFB runs) to monitor participants' experience of (i) tenderness, anguish and neutral states (from 1 = very mild to 5 = very intense), (ii) how useful they found the emotion regulation strategies (from 1 = very little to 5 = very useful), (iii) how easy they found to use the virtual environment BCI (from 1 = extremely difficult and 5 = extremely easy), (iv) how easy they found to change the color of the virtual environment BCI during rtfMRI-NFB (from 1 = extremely difficult and 5 = extremely easy), (v) fatigue (from 1 = not at all and 5 = extremely) and (vi) focus (from 1 = not at all and 5 = extremely).

After MRI, we administered the *Emotion Regulation Questionnaire* (39), *Satisfaction with Life Scale* (41), and *Body Vigilance Scale* (40). After the follow-up assessment (end of day 2), participants were administered VAS scales to rate (from 1 = not at all, to 10 = extremely) how much the music excerpts evoked ten different positive and negative emotional states including anguish and tenderness, enchantment, transcendence, strength, serenity/peace, joy, nostalgic, sadness and tension.

### Off-line statistical analyses

#### Behavioral data

Participants' strategies to up-regulate tenderness, anguish and neutral states were qualitatively described. Chi-square and *T*-tests were run to compare participants' sex, age, questionnaire and VAS data between sites.

Repeated measures ANOVAs were run using site as a between-subject factor (site 1 and site 2) and assessment time as a repeated measure (pre MRI and post MRI) to assess the effects of site and NFB task on BDI, STAI and PANAS scores.

Repeated measures ANOVAs were performed using site as between-subject factor and MRI run as repeated measure (one training run and three rtfMRI-NFB runs) to assess their effect on participants' experiences during rtfMRI-NFB (i.e., emotion intensity, how useful they found their emotion regulation strategies and to change the color of the virtual environment BCI during rtfMRI-NFB).

Two linear mixed models tested the effect of the three rtfMRI-NFB runs, the method to compute brain activity in real time (SVM and ROI) and assessment site, on the change in the color of the virtual environment BCI during rtfMRI-NFB (i.e., the degree of orange saturation for the tenderness condition, and purple saturation during the anguish condition).

Finally, *t*-tests compared emotion ratings of the music excerpts between the anguish tenderness and neutral conditions. We used IBM SPSS Statistics v22.0.0.0.

#### MRI data processing

MRI data was processed offline using Statistical Parametric Mapping 12 software v6470 (SPM12; www.fil.ion.ucl.ac.uk/spm).

##### Offline MRI data pre-processing and first level analysis

Offline MRI data Preprocessing included realignment, slice timing, normalization using T1-weighted images and smoothing. We corrected first level analysis results for artifacts, outliers and motion correction parameters. First level MRI data were quality checked to identify problematic volumes (e.g., distortions, movements, etc.) visually using the Medical Image Processing, Analysis, and Visualization tool (MIPAV, https://mipav.cit.nih.gov/), and automatically to identify artifacts of movements over 3 mm for translation and 0.02 radians for rotation via the Artifact Detection Tools (ART; http://www.nitrc.org/projects/artifact_detect). We used as a high-pass filter the double of the max length time between the same stimuli, 456 s for SVM and 152 s for ROI.

##### Offline fMRI fixed effect group analysis.

We first run t-contrasts to examine how brain activity was affected by emotion type (*Tenderness* vs. *Anguish* and *Anguish* vs. *Tenderness*), rtfMRI-NFB method (SVM and ROI) and assessment site. To gain power to detect these effects in a small group of participants, we analyzed the rtfMRI-NFB data using a fixed effects model (42, 43). First, to test our hypotheses on the engagement of specific ROIs during the conditions tenderness and anguish, SVC were applied using the ROIs from the ROI based rtfMRI-NFB method with a whole-brain voxel threshold of *p* < 0.005, uncorrected. Second, to test whether the hypothesized regions were still implicated using a more conservative approach, we ran analyses of rtfMRI-NFB data using FWE correction at a whole brain level with *p* < 0.05.

## Results

Results are summarized starting with sample demographic characteristics and questionnaire data (e.g., mood, anxiety, personalized strategies), followed by a description of the ratings of task variables (e.g., rtfMRI-NFB task, intensity of the emotions at the end of the rtfMRI-NFB runs, experience of the BCI, audio tracks) and brain activity patterns for the rtfMRI-NFB conditions of tenderness and anguish (i.e., small volume FWE corrected results), followed by whole brain FWE results for the whole group and separately by experimental site and rtfMRI-NFB method (ROI and SVM).

### Sample characteristics

Sample demographic and questionnaire data are overviewed in Table 1. We recruited eight 23 to 28-year-old participants separately from two sites (Site 1, *N* = 4; Site 2, *N* = 4). The groups from the two sites were matched by age, sex and scores for depression, positive affect, satisfaction with life, emotion regulation and body vigilance during MRI.

**Table 1:** Summary of demographic and questionnaires data by Site 1 and site 2.

	**Site 1**		**Site 2**		**T (df), p**	
	**Pre-MRI**	**Post-MRI**	**Pre-MRI**	**Post-MRI**		
N(females)	4 (1)		4 (2)		*X* = 1.07, *df* = 1,14, *p* = 0.30	–
age	24.75 (1.58)		25.75 (1.39)		*T* = −1.34, *df* = 1,14, *p* = 0.20	–
BDI	2.12 (2.80)	1.88 (2.64)	3.88 (3.18)	3.38 (2.61)	*F* = 1.38, *df* = 1,14, *p* = 0.26	*F* = 2.03, *df* = 1,14, *p* = 0.18
STAI	45.38 (8.77)	43.88 (6.75)	27.88 (4.39)	29.50 (4.17)	***F* = 28.20, *df* = 1,14, *p* <0.001**	*F* = 0.04, *df* = 1,14, *p* = 0.95
Positive Affect	26.13 (9.03)	24.13 (9.75)	36.00 (6.16)	34.75 (7.56)	*F* = 6.45, *df* = 1,14, *p* <0.05^*^	*F* = 3.80, *df* = 1,14, *p* = 0.07
Negative Affect	3.75 (3.92)	3.13 (4.12)	12.13 (1.73)	13.13 (2.10)	*F* = 37.79, *df* = 1,14, *p* <0.001^*^	*F* = 0.14, *df* = 1,14, *p* = 0.13
ERQ Reappraisal	–	34.63 (4.87)	–	30.70 (4.03)	*T* = 1.74, *df* = 1,14, *p* = 0.11	–
Suppression	–	13.13 (3.40)	–	13.75 (3.66)	*T* = −0.35, *df* = 1,12, *p* = 0.73	–
Satisfaction with life	–	27.71 (3.04)	–	29.38 (4.30)	*T* = −0.85, *df* = 1,13, *p* = 0.41	–
Body vigilance	–	20.38 (6.99)	–	28.75 (12.03)	*T* = −1.70, *df* = 1,14, *p* = 0.11	–

*Site 1 = D'Or Institute for Research and Education, Rio de Janeiro; Site 2 = Monash Biomedical Imaging, Monash University, Melbourne; Mean (standard deviation values); ERQ, emotion regulation questionnaire (44); satisfaction with life scale (41); Body vigilance scale (40); BDI, Beck depression inventory (36); STAI, state and trait anxiety inventory (37); PANAS, positive affect and negative affect scale (45)*.

* *These results did not survive Bonferroni correction for multiple comparisons. Bold fonts indicate p < 0.05*.

### Personalized strategies to achieve the target emotions

Participants' emotion regulation strategies varied. Neutral emotional states were achieved by recalling non-salient personal memories and imagined trivial scenarios and by mentally repeating neutral mantras (e.g., the world is round/full of water/a planet, I am laying in the MRI scanner, I am laying down, the leaves move).

Tenderness states were achieved and maintained via strategies including thoughts of loved partners, friends, young relatives or pets, pleasant memories (e.g., of own childhood, playing with nieces/nephews, memorable moment with loved partner and friends), and via repeating mantras (e.g., the world is beautiful/love/safe/generous/has love everywhere; people love each other; people are nice; friends are special; love is all that matters/is everything, I am love, affection exists).

Anguish states were experienced via recalling memories and imagining negative scenarios (e.g., illness/death/arguments with close people, cruelty to pets, war, stuck in the MRI room/in a fire/in water drowning/in own mind) and via repeating unpleasant mantras (e.g., “the whole world is dying”).

### Ratings of emotions and of neurofeedback task variables

**Figure 4:**
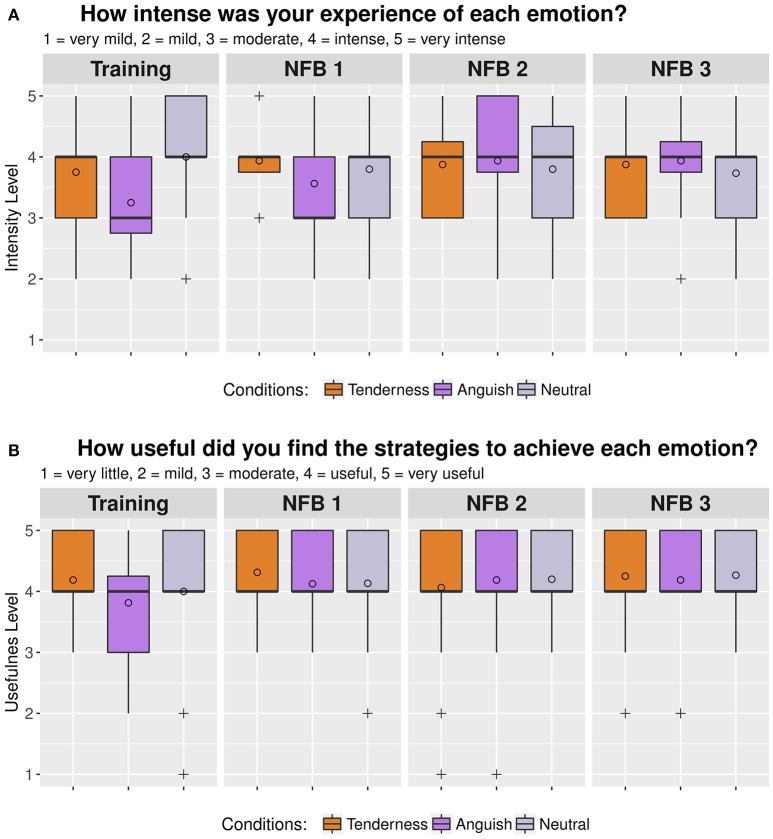
Box plot of self-reported rating measured in eight participants over two consecutive assessment days immediately after each of the four MRI runs. **(A)** self-reported intensity of emotions during neurofeedback and **(B)** self-reported usefulness of strategies to up regulate emotions. The circles represent the mean values and the crosses represent outliers.

Supplementary Table 1 and Figure 4 overview the effect of NFB run (1-to-3) and site (Site 2 vs. Site 1) on emotion regulation and rtfMRI-NFB variables.

The intensity of the emotions during the rtfMRI-NFB task was rated as “moderately intense” for anguish and between “moderately intense” and “intense” for tenderness. The emotion intensity ratings were affected by site (Site 2 > Site 1) but participants experienced a similar intensity of emotions across the rtfMRI-NFB runs.

All participants found their strategies to be “moderately useful,” across the rtfMRI-NFB runs (i.e., non significant effect of rtfMRI-NFB run) and this was affected by site (Site 2 > Site 1).

The virtual environment BCI was rated as “moderately” easy to use during tenderness, anguish and neutral conditions across participants from the three rtfMRI-NFB runs and the two sites, through the neutral condition was affected by site (Site 1 > Site 2).

Participants rated that it was “neither difficult nor easy” to detect the color change in the virtual environment BCI across all rtfMRI-NFB runs, but more markedly in one site (Site 1 > Site 2).

Finally, there was an effect of site on the level of tiredness (Site 1 > Site 2) and focus (Site 2 > Site 1), and both were “moderate” across all rtfMRI-NFB runs.

### Rating of the audio tracks used during the conditions “anguish” and “tenderness”

Participants' ratings of the emotions induced by the audio tracks during rt-fMRI NFB (anguish and tenderness conditions) are overviewed in Supplementary Table 2. The music tracks used elicited significantly higher levels of tenderness and positive emotions (i.e., enchantment, transcendence, strength, serenity, joy) and trend-like higher level of nostalgia, potentially as participants' evoked past experiences. The music tracks used during anguish elicited significantly higher levels of anguish, sadness and tension.

### Level of real time color change of the virtual environment in the BCI during rtfMRI-NFB

**Figure 5:**
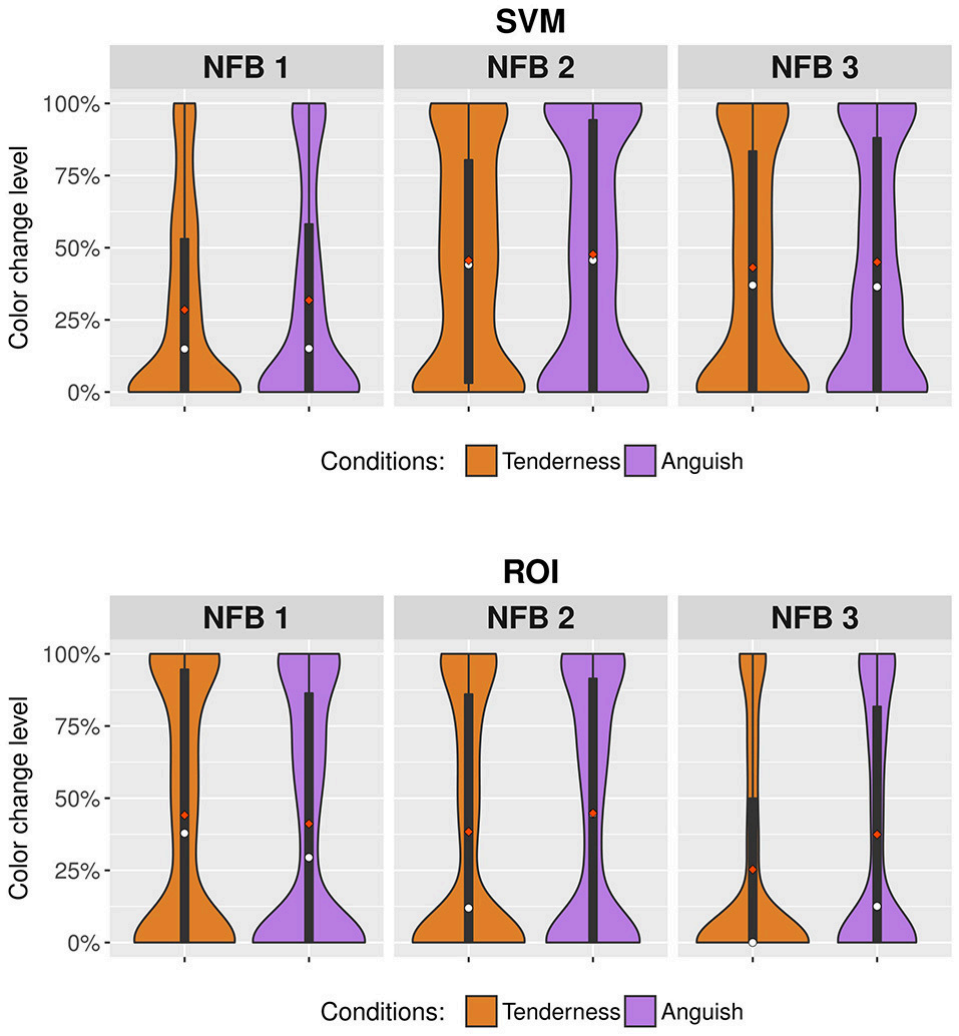
Violin plots showing changes in the color of the virtual environment BCI during rtfMRI-NFB. Results from the rtfMRI-NFB runs are shown for the tenderness condition (orange plots) and anguish condition (purple plots) by the rtfMRI-NFB methods which include SVM = support vector machine and ROI = region of interest. The width of the violin plots changes according to the concentration of the results for specific levels of color change. Mean values are illustrated in red dots, and median values in white dots.

Figure 5 shows the change in the color of the virtual environment BCI during rtfMRI-NFB using two distinct NFB methods. The change in color of the BCI was significantly affected by rtfMRI-NFB runs (Tenderness: *F* = 7.53, *df* = 2, *p* = 0.001, Anguish: *F* = 6.78, *df* = 2, *p* = 0.001), assessment site (Site 2 > Site 1, Tenderness: *F* = 27.16, *df* = 1, *p* < 0.001, Anguish: *F* = 4.17, *df* = 1, *p* = 0.041) and rtfMRI-NFB method (ROI > SVM for Site 1*: F* = 34.132, *df* = 1, *p* < 0.001; SVM > ROI for Site 2: *F* = 21.03, *df* = 1, *p* < 0.001). Results separated by site reveal similar patterns and are shown in Supplementary Figure 5.

### Offline fMRI analyses on brain activity during rtfMRI neurofeedback

Offline fMRI data analysis of rtfMRI-NFB runs confirm that participants successfully recruited the hypothesized areas and additional brain regions at a group level. Brain activity within the septo-hypothalamic ROI during tenderness rtfMRI-NFB trials was first examined using SVC FWE correction (*p* < 0.05, *k* = 5).

The tenderness rtfMRI-NFB condition significantly engaged the predicted septo-hypothalamic area (*k* = 48, *T* = 5.19, *x* = 3, *y* = 14, *z* = −7). The same results emerged when repeating the analyses with SVC FWE correction separately by site (Site 1, *k* = 31, *T* = 3.72, *x* = 0, *y* = 14, *z* = −10; and Site 2, *k* = 5, *T* = 3.99, *x* = −9, *y* = 8, *z* = −16) and separately by NFB method (SVM: *k* = 77, *T* = 4.62, *x* = 0, *y* = 11, *z* = −13, and ROI: *k* = 7, *T* = 4.13, *x* = 3, *y* = 14, *z* = −7).

**Figure 6:**
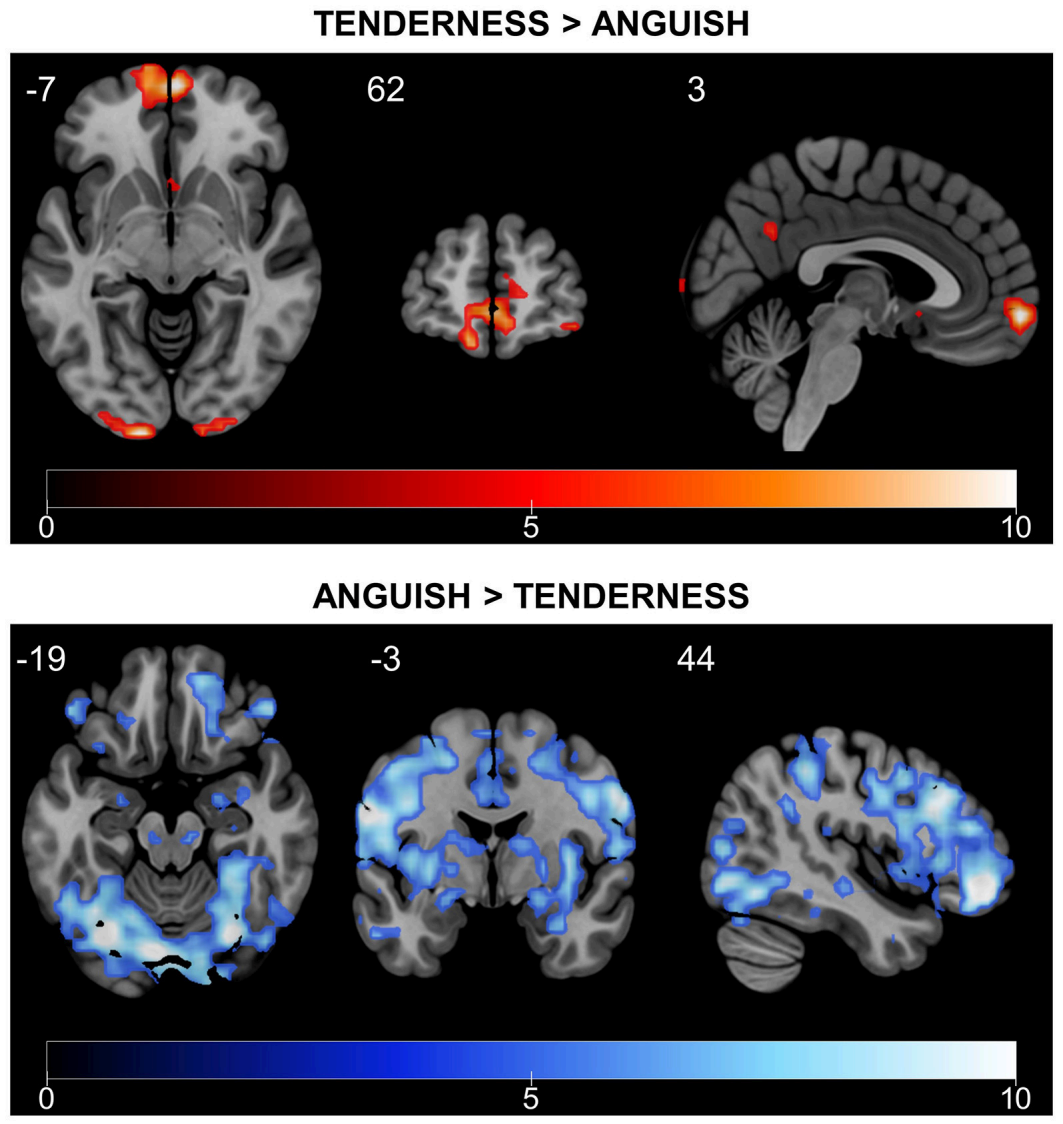
Differential rtfMRI-NFB-related brain responses for tenderness and anguish conditions. *Tenderness* vs. *anguish* rtfMRI-NFB recruited the septo-hypothalamic area, the frontal pole and the precuneus. *Anguish* vs. *tenderness* rtfMRI-NFB recruited a more widespread network including the superior/middle frontal cortex, frontal pole, parietal cortical regions, temporal regions (middle and inferior) and other regions (lateral occipital, central operculum, cerebellum). Results were estimated across all participants (*N* = 16, two scans per subject) via fixed-effect analysis and whole-brain FWE correction with *p* < 0.05, *T* > 4.716).

We also examined brain activity during the rtfMRI-NFB task with a whole brain approach and FWE correction (*p* < 0.05, *k* = 5) (Figure 6 and Tables 2, 3). The results also show engagement of the septo-hypothalamic area and the frontal pole (including medial orbitofrontal regions), the temporal pole and the precuneus. Similar results emerged when examining the rtfMRI-NFB data separately by site (Supplementary Figure 2 and Supplementary Table 3) and by NFB method (Supplementary Figure 3 and Supplementary Table 4). Notably, the same patterns emerged in individual participants' activation maps, shown in Supplementary Figure 4.

**Table 2:** Overview of local maxima for brain activity during *Tenderness versus Anguish* neurofeedback conditions, across the whole brain.

**Brain area**	**Local maxima**		**MNI Coordinates**			**Brodmann Area**
	**Extent**	***t*-value**	***x***	***y***	***z***	
Occipital pole	129	12.662	−6	−103	14	17
	129	10.780	−15	−103	−4	17
	129	5.899	−33	−94	−7	18
	51	12.373	21	−100	14	17
	21	7.270	15	−103	−7	17
	8	5.160	3	−100	8	17
Frontal pole	193	10.633	3	62	−7	10
	6	7.324	39	59	−13	47
	26	6.699	−24	41	50	9
Frontal medial cortex	14	6.594	−9	44	−13	11
Precuneus	21	6.167	3	−55	32	23
Middle frontal gyrus	7	6.111	−42	23	53	9
	8	6.022	33	29	56	8
Temporal pole	5	5.062	36	20	−37	38
^*^Septo-hypothalamic area	3	5.189	3	14	−7	25

*Table shows all local maxima separated by > 20 mm, surviving threshold of p < 0.05 (FWE-corrected), t > 4.7160, df = 18,548 minimum extent = 5. Regions were automatically labeled using the HarvardOxford-maxprob-thr0 atlas. x, y, and z = Montreal Neurological Institute (MNI) coordinates in the left-right, anterior-posterior, and inferior-superior dimensions, respectively*.

* *subcallosal region*.

**Table 3:** Overview of local maxima for brain activity during *Anguish versus Tenderness* neurofeedback conditions, across the whole brain.

**Brain area**	**Local maxima**		**MNI Coordinates**			**Broadmann Area**
	**Extent**	***t*-value**	***x***	***y***	***z***	
Occipital cortex, lateral left^*^	18,866	15.627	−21	−82	29	19
Dorsolateral PFC^*^		15.181	−39	44	−13	47
Fusiform cortex, temporal occipital, left^*^		13.855	−30	−61	−10	37
Frontal pole	36	7.782	6	71	11	10
Middle temporal gyrus, anterior	18	7.518	−54	−4	−31	20
Posterior cingulate gyrus, left^*^	25	6.515	0	−40	8	29
Frontal pole	11	6.401	−12	65	8	10
Brain-stem	8	5.809	−6	−25	−19	35
Inferior temporal gyrus, anterior	6	5.755	48	2	−34	20
Subcallosal cortex	5	5.564	−9	29	−22	11
Superior temporal gyrus, anterior	8	5.508	63	−7	−1	41
Orbitofrontal cortex	9	5.461	−21	32	−19	11

Table shows all local maxima separated by >20 mm, surviving threshold of p < 0.05 (whole brain FWE-corrected), t > 4.7160, df = 18548, minimum extent = 5. x, y, and z = Montreal Neurological Institute coordinate in the left-right, anterior-posterior, and inferior-superior dimensions, respectively. Regions were automatically labeled using the HarvardOxford-maxprob-thr0 atlas and regions with an

* *were the nearest location of activations using the same atlas*.

Brain activity during anguish rtfMRI-NFB trials was first examined using a small volume FWE correction (*p* < 0.05, *k* = 5). The right amygdala area was robustly engaged in the whole sample (*k* = 214, *T* = 8.43, *x* = 24, *y* = −10, *z* = −13), and also when examining data separately by site (Site 1: *k* = 42, *T* = 5.24, *x* = 33, *y* = −7, *z* = −7, and Site 2: *k* = 236, *T* = 8.99, *x* = 30, *y* = −7, *z* = −22) and by NFB method (SVM: *k* = 176, *T* = 6.43, *x* = 33, *y* = −4, *z* = −7, and ROI: *k* = 174, *T* = 6.98, *x* = 24, *y* = −10, *z* = −13).

*Anguish* vs. *Tenderness* results (see Figure 6 and Table 3) during rtfMRI-NFB using a whole brain approach with FWE correction (*p* < 0.05, *k* = 5) show the recruitment of the amygdala, frontal regions (i.e., polar, superior and middle areas), parietal regions (i.e., angular and supramarginal gyri, juxtapositional lobule), temporal (middle and inferior) and other cortical regions (lateral occipital, central operculum, cerebellum). Similar though weaker pattern of brain activity emerged when examining the results separately by site (Supplementary Table 2) and by rtfMRI-NFB method (Supplementary Table 3). Finally, the same patterns were apparent in individual participants' activation maps shown in Supplementary Figure 5.

## Discussion

We provide for the first-time proof of concept and demonstrate feasibility of the implementation of rtfMRI-NFB using virtual environment BCI and music to elicit and measure the neural correlates of specific, complex emotional states. In line with our expectations, real-time up-regulation of tenderness engaged the septo-hypothalamic area and other regions previously implicated in positive affiliative emotions (i.e., medial frontal cortex and temporal pole, precuneus). Additionally, online up-regulation of anguish recruited a widespread network of regions ascribed to negative affect, including the amygdala, dorsolateral prefrontal and additional regions. These effects were corroborated by individual brain activation maps, and by group activation maps across the two experimental sites and the two NFB methods, as well as by self-reported emotions experienced during NFB. Our findings preliminarily validate the notion that individuals can experience powerful emotional states and recruit relevant brain networks in real time using a novel multisensory rtfMRI-NFB tool comprising a virtual environment BCI.

Up-regulation of tenderness states recruited three clusters of brain areas previously implicated in positive affiliative emotions. These include the septo-hypothalamic region, the frontal pole, the medial orbitofrontal cortex, the temporal pole and the precuneus. The validity of our findings on tenderness-related brain networks is corroborated by the involvement of these regions in previous fMRI work on affiliative emotions (11, 22, 25) and their specificity to the experience of tenderness is supported by participants' reports that their tenderness states increased/were sustained during the NFB tenderness condition.

We show that the septo-hypothalamic region was key for the experience of tenderness states. This is consistent with our previous rtfMRI-NFB study also targeting tenderness (11). Yet, this region may be ascribed to affiliative emotions generally including but not limited to tenderness [e.g., empathy, compassion, guilt and others (23)]. Indeed, previous fMRI experiments targeting positive affiliative emotions other than tenderness implicate the septo-hypothalamic region (11, 23, 25). Also, lesion evidence shows abnormal prosocial affect in patients affected by lesions of the septo-hypothalamic area (13) and by neurological disorders (i.e., frontotemporal dementia) compromising this area (46, 47).

rtfMRI-NFB during the tenderness condition recruited - in addition to the septo-hypothalamic area - the medial prefrontal (i.e., frontal medial, middle frontal gyrus), temporal and parietal regions (i.e., precuneous). This is consistent with neurobiological evidence and theories of affiliative emotions, suggesting that our rtfMRI-NFB study was successful. Yet, we failed to detect activity in the subgenual/ventral cingulate cortices (22, 48), which have been implicated in the neurobiology of additional affiliative emotions (e.g., compassion and guilt). This discrepancy may be explained by the different cognitive demands required in the current rtfMRI-NFB study and previous fMRI studies (11, 23–27, 47), particularly as this was the only study to use personalized strategies to increase and maintain the intensity of the emotions and to use emotions to voluntary regulate brain activity in real time. Given the pilot nature of our study and the many elements included in the experiment (e.g., rtfMRI-NFB, virtual environment BCI, real time fMRI, mood induction, personalized strategies, audio tracks and others) further assessments are required to determine specific methodological factors in our study played a role in the partially discrepant findings with the literature to date.

rtfMRI-NFB during the anguish conditions, recruited a much more widespread network of regions comprising the amygdala and fronto-parietal, temporal and other cortical regions. The recruitment of the amygdala is consistent with our hypothesis and previous fMRI evidence on negative affect (49–52). Our results mirror those from previous fMRI studies on negative emotions that also implicate temporal (51), prefrontal (53–56), frontal polar (57, 58), and parietal regions (54). The overlapping brain networks between our study and previous work on negative affect suggest that our rtfMRI-NFB protocol successfully recruited the target brain network. Future work contrasting distinct complex negative emotions is required to clarify if this network is ascribed to anguish specifically rather to negative emotions that are intense, arousing and potentially threatening including but not limited to anguish—such as fear, emotional pain and anxiety (57, 59, 60).

The anguish condition engaged a widespread pattern of brain regions. Additional higher order cognitive control brain areas may have been recruited due to the complex cognitive demands associated with the task, including attention control, evaluation and voluntary regulation of negative emotions, cognitive efforts required for maintaining complex emotions (54, 57, 61–67). Indeed, participants reported to habituate quickly to anguish states, as the thoughts that originally elicited anguish, were no longer effective after a short period. Participants used additional cognitive strategies to maintain anguish states, including to think of new memories and thoughts and imagine other scenarios.

We did not directly compare SVM and ROI rtfMRI-NFB methods given the pilot nature of the study and the fundamentally distinct measures of brain activity. Yet, we explored whether the hypothesized networks were recruited more robustly using either method. Both ROI and SVM rtfMRI-NFB methods recruited similar networks and showed comparable accuracy rates. This is interesting as SVM has been recognized to be superior to ROI in handling low signal to noise in areas susceptible to artifacts, decoding complex brain states with high sensitivity and accounting for individual variability (68, 69).

This issue cannot be resolved in this pilot study as it relies on a small sample size. Yet, our goal was to deliver a proof of concept for a novel real-time fMRI neurofeedback approach and software tool that can be used in future studies aiming to test mechanistic or clinical hypotheses, and not to provide definitive evidence for the superiority of ROI over SVM approaches (or vice-versa) or to establish unequivocally the role of fMRI neurofeedback in helping volunteers achieve emotional states more efficiently. This pilot methodological study demonstrates the feasibility of this novel neurofeedback method and software tool and its usability across research centers and teams to provide real-time emotional neurofeedback using virtual scenarios, employing either ROI or SVM-based metrics.

### Limitations

Our study presents some important limitations. First, while self-reported emotions and previous work corroborated the patterns of brain activity, the lack of an active control condition (e.g., sham feedback from a separate region, artificially created or from another dataset) prevents the understanding of whether confounding variables have driven our results (e.g., rtfMRI-NFB, task practice, arousal, general intentional/motivation factors, others). Nonetheless, we would like to emphasize that this is a proof of concept study not aimed at showing differences between real and sham conditions, but at providing key insights on the technological implementation of multimodal, fMRI-NFB using a virtual environment as BCI and its feasibility for conducting single-subject studies.

Second, we did not use a rtfMRI-NFB transfer run to examine if participants had learned or could transfer the skills outside the MRI environment. We prioritized to acquire brain data from rtfMRI*-NFB* to test our new platform (4).

Third, we did not measure emotion subjectively in a continuous fashion, but at the end of each neurofeedback run. Our pilot real-time fMRI neurofeedback study did not aim to test statistically significant effects in emotional learning/enhancement across runs. Yet, our study provides evidence for feasibility along with guidelines, a protocol, and a free software tool that enables other researchers to conduct (emotional) fMRI neurofeedback integrated with a VR/game platform.

Fourth, we used a set of matched audio tracks for the conditions of tenderness and anguish to minimize systematic differences due to using different music tones and rhythms. However, the valence of the different audio tracks may have engaged distinct neural networks possibly confounding our results (70). We used the same audio tracks for all participants and these may have not helped all equally to achieve the target emotions, due to inter-individual differences in taste in music, personalities and other psychological variables. Personalized audio tracks may have been more effective in eliciting powerful and individually salient emotional states. However, participants' ratings of the audio tracks show that these induced the target tenderness, anguish and other positive and negative emotions.

Additionally, participants used different strategies to experience different emotions or the same emotion over time, which were qualitatively described and not controlled for in the brain activity analyses. The use of discrepant strategies may have biased brain activity (i) during NFB tenderness and anguish blocks, which were derived relative to the previous neutral blocks (ii) measured post-acquisition when contrasting tenderness and anguish. On the other end, personalized strategies ensured that each individual found the best way to feel valid emotional states. Our findings from participants' rating of their emotion intensity and the consistent patterns of brain activity in individual brain activation maps suggest that the target neurobehavioral states were achieved despite—or because of—personalized emotion regulation strategies.

Patterns of brain activity may differ from subject to subject or from session to session. This differential responsiveness means that the fixed-effect statistical analyses may not be appropriate when trying to generalize inferences (42). In our case, this analysis fits well since we are working with a restricted group that has been trained to perform the emotional task, and making inferences to an additional group of subjects was not our goal (43). Instead, providing robust results at the individual subject level is an important step toward clinical applications.

### Future directions and conclusions

This novel rtfMRI-NFB platform is a promising tool for future experiments and interventions, particularly as the virtual environment BCI and musical excerpts can be individually customized to maximize participant's engagement. This platform can be changed or replaced by other multisensory approaches (tactile, auditory, sensory, etc.) according to specific experimental/clinical intervention needs, and is compatible with other platforms routinely used in experimental psychology and neuroscience research (e.g., MATLAB, EPrime, Presentation, Python, R and others). Participants successfully and voluntarily shifted from a brain pattern of intense negative emotions to a pattern of positive affiliative emotions. Our findings may contribute to the understanding of the neurobiological mechanisms of psychological interventions that boost positive affiliative emotions—such as compassion focused therapies (71) and loving-kindness meditation—and neuroplasticity (72, 73). Our study may inform the development of non-invasive, brain-based therapies that boost positive affiliative emotions—possibly even via hyper scanning—that have beneficial effects for a range of psychopathologies—e.g., depression, borderline personality disorder, psychopathy, and others.

In sum, we validated a novel rtfMRI-NFB protocol and instrument using a multimodal stimulation for future experimental and clinical intervention. We warrant replication studies using active control conditions [e.g., sham rtfMRI-NFB, biofeedback, psychotherapy, pharmacotherapy, physiotherapy, or other physical interventions (4)].

Future developments for rtfMRI-NFB platforms incorporating virtual environments as BCI may include providing feedback on different properties of brain functions including but not limited to connectivity and multiple ROIs concurrently (both possible with the Friend Engine platform), and tailoring rtfMRI-NFB tasks with multi-sensory BCIs to the needs of the individual and target population in large samples (e.g., videogame like interface for children, feared stimuli in participants with phobias, mannequins that can move with brain activity in patients with stroke with impaired motor function), to identify the characteristics of those who respond best and least and inform evidence based interventions. Our results warrant further rtfMRI-NFB studies using personalized interfaces in large cohorts to examine the therapeutic potential of rtfMRI-NFB in clinical samples, and its ability to enhance cognitive and emotional wellbeing in normative populations.

## Data availability

The datasets used in the current study are available from the corresponding author on reasonable request.

## Author contributions

VL and BM led the study execution, protocol setup, data analysis, and the writing of all the aspects of the manuscripts from start to completion. RB led the stimulus and neurofeedback setup in the D'Or laboratory in Rio and provided assistance in setting up the neurofeedback platform at Monash University in Melbourne. CS contributed to data collection and led the neurofeedback software setup in the Melbourne site. MY and CT-C advised on the running of the project and revised the manuscript. JM advised on the running of all the aspects of the study, overviewed the experimental protocol setup and the running of the fMRI analyses; and contributed to the writing of the first and other drafts of the manuscript.

### Conflict of interest statement

The authors declare that the research was conducted in the absence of any commercial or financial relationships that could be construed as a potential conflict of interest.
