# Supplementary material for: Emotion Regulation Using Virtual Environments and Real-Time fMRI Neurofeedback
Source: Front Neurol. 2018 Jul 24;9:390. doi: 10.3389/fneur.2018.00390 (PMC6066986; doi:10.3389/fneur.2018.00390)
Supplement: Supplementary file 1 [file Data_Sheet_1.docx]

Supplementary Material

Emotion regulation using virtual environments and real-time fMRI neurofeedback

Valentina Lorenzetti, Bruno Melo, Rodrigo Basílio, Chao Suo, Murat Yücel, Carlos Julio Tierra-Criollo, Jorge Moll^*^

*** Correspondence:** Dr. Jorge Moll: [jorge.moll@idor.org](mailto:jorge.moll@idor.org)

# Supplementary Data

**Supplementary Video 1.** The video illustrates the color change of the virtual environment interface and the corresponding change in brain activity. Cross-hairs are positioned in the septal-hypothalamic area during Tenderness trials and the preceding Neutral trials, and in the amygdala during Anguish trials and the preceding Neutral trials. The video starts with a Neutral trial with a baseline natural virtual environment color, followed by an Anguish trial with the color of virtual environment turning to purple (46”), a Neutral trial and then a Tenderness trial with the color of the virtual environment turning to orange (46”). For simplicity, the duration of the Neutral trials in this video is shorter than that used in the actual experiment.

# Supplementary Figures and Tables

## Supplementary Figures

**
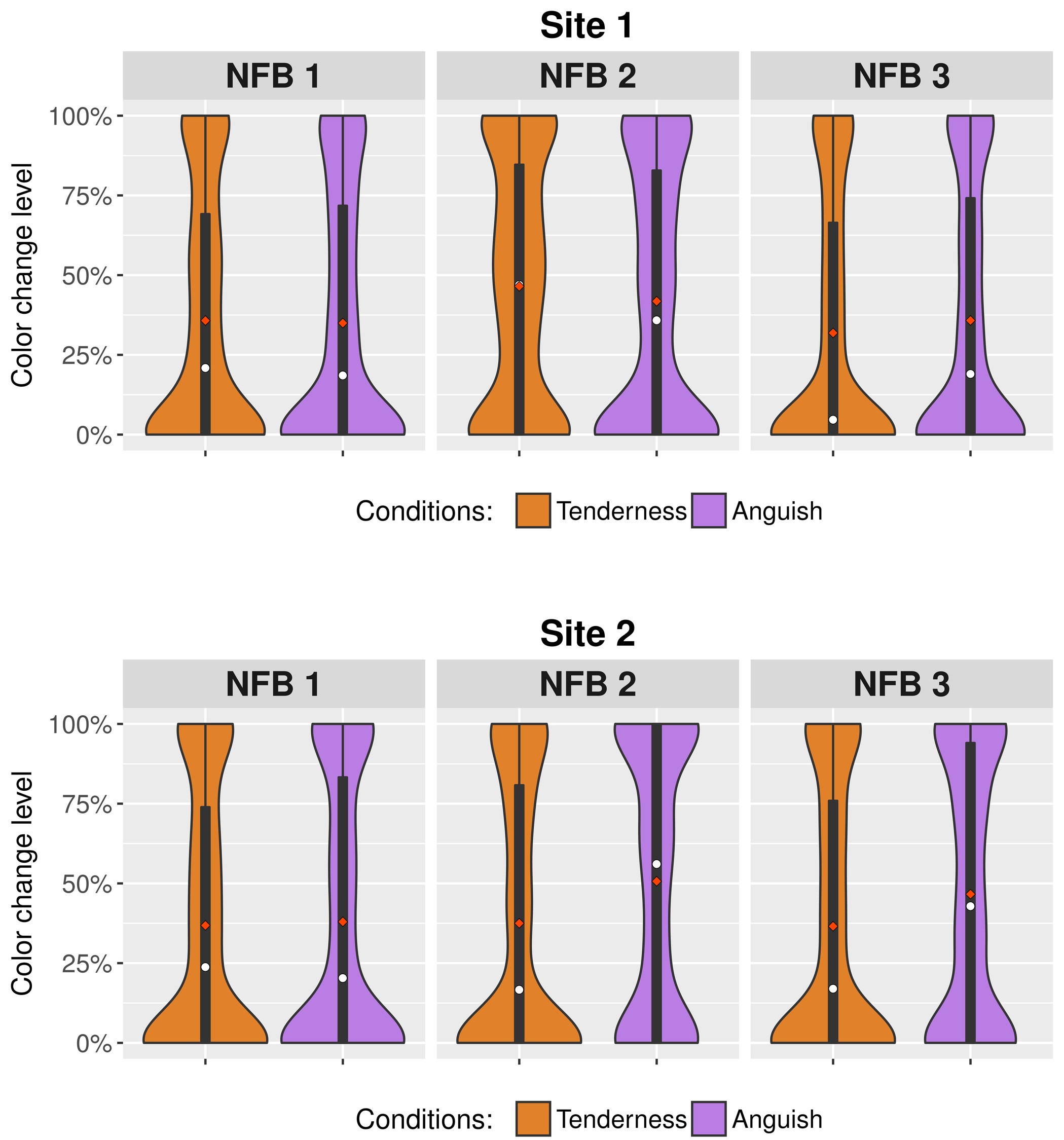
**

**Supplementary Figure 1**. Violin plots showing the level of change of the color of the virtual environment neurofeedback interface during Tenderness trials (orange plots) and Anguish trials (purple plots). The level of color change ranged from 0% to 100% and is summarized by recruitment site, with Site 1 (D’Or Institute for Research and Education, Rio de Janeiro) and (B) Site 2 (Monash Biomedical Imaging, Monash University, Melbourne). Red dots represent mean values, white dots represent median, the width of the plot varies to show the distribution of data.

**
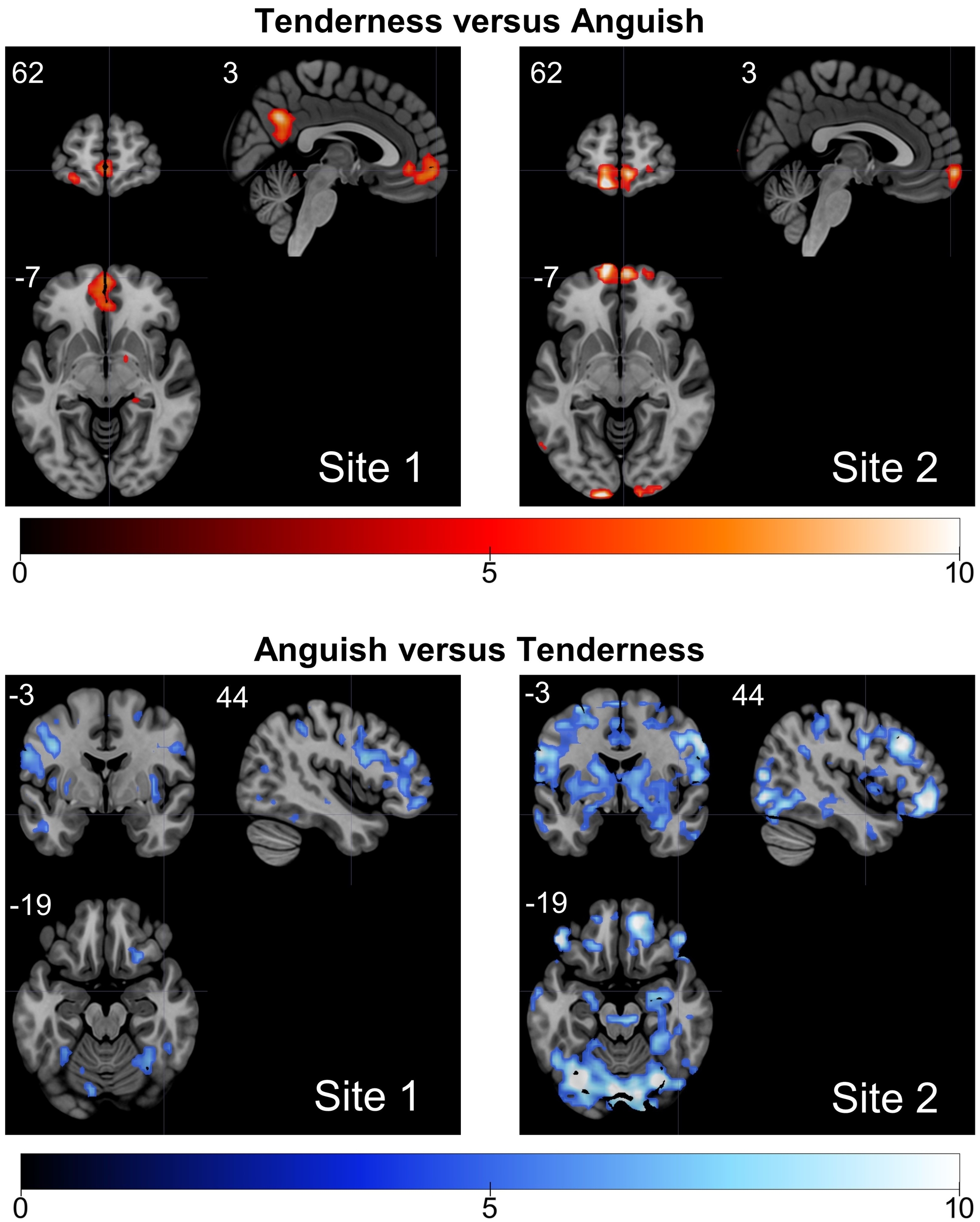
**

**Supplementary Figure 2.** Color maps of brain activity during Tenderness versus Anguish trials (red-to-yellow) and Anguish versus Tenderness (blue-to-light blue) representing T-values segregated by Site 1 (D’Or Institute for Research and Education, Rio de Janeiro) and Site 2 (Monash Biomedical Imaging, Monash University, Melbourne) using a fixed-effect analysis, with whole-brain FWE correction, p < 0.05 (T > 4.716).


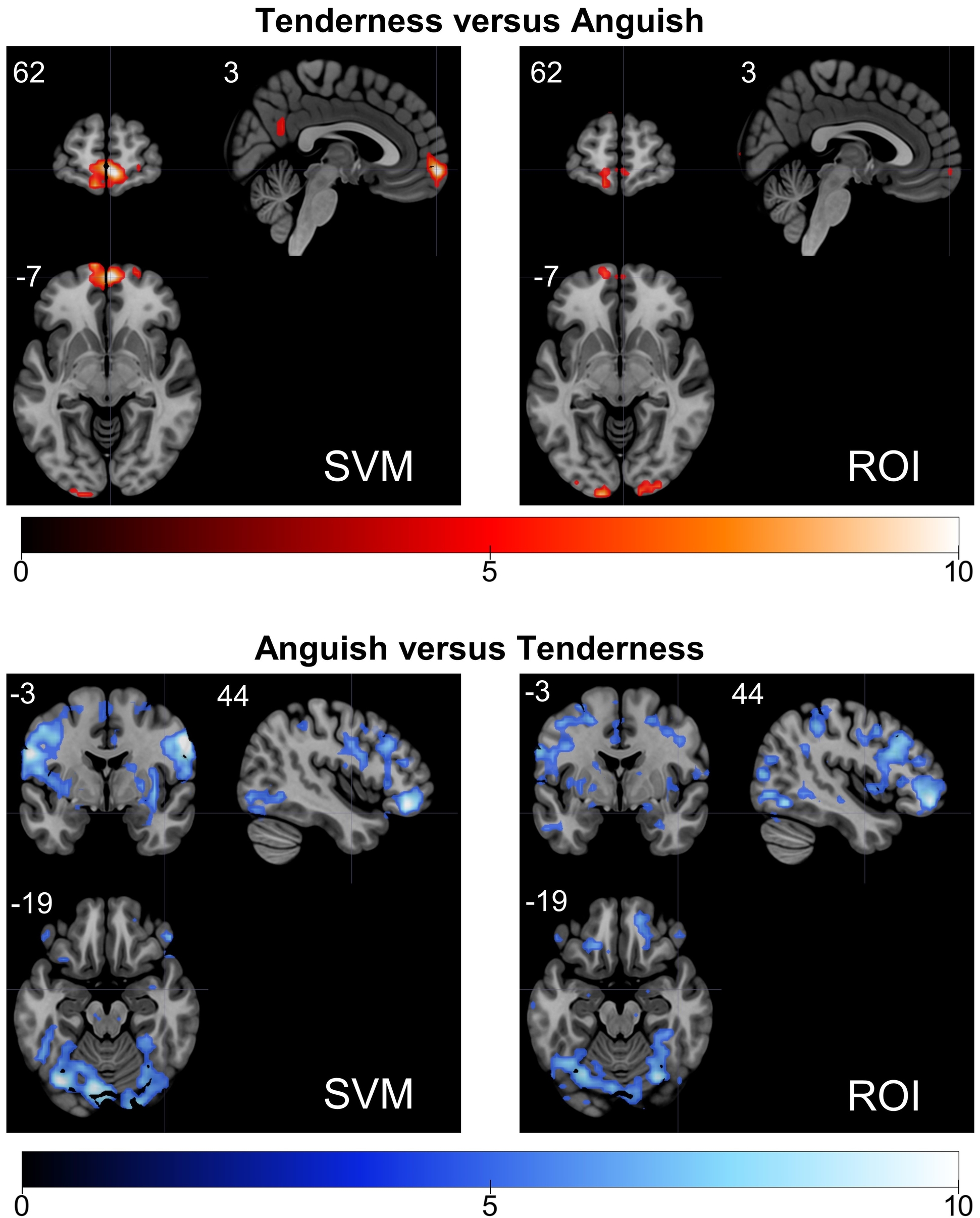


**Supplementary Figure 3.** Color maps of brain activity during Tenderness versus Anguish trials (red-to-yellow) and Anguish versus Tenderness (blue-to-light blue) representing T-values segregated by NFB method (ROI and SVM) using a fixed-effect analysis, with whole-brain FWE correction, p < 0.05 (T > 4.716).


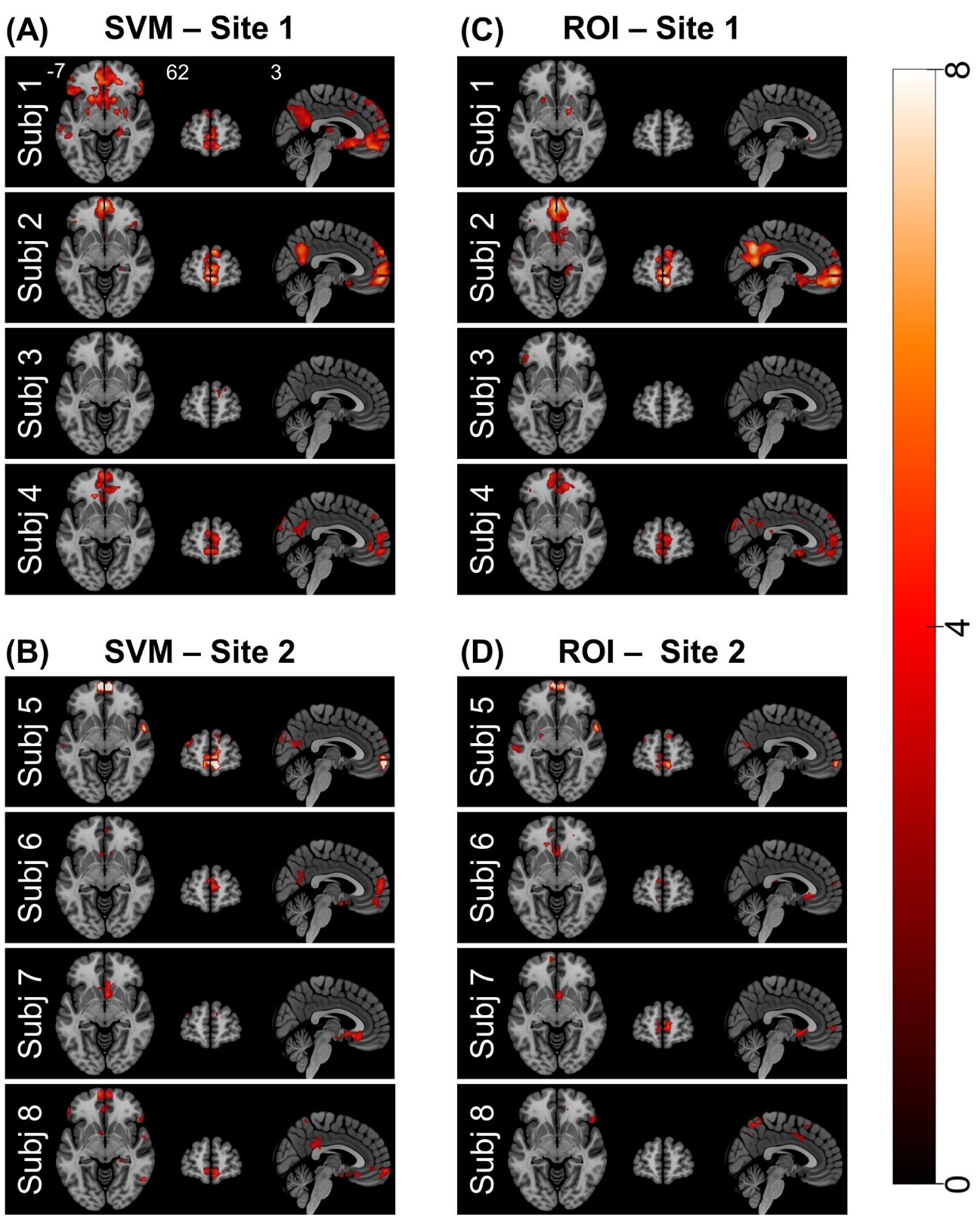


**Supplementary Figure 4.** Individual patterns of brain activity during neurofeedback Tenderness > Anguish trials using two distinct neurofeedback methods including SVM = support vector machine (left panels) and ROI = region of interest (right panels). The top panels show results for both SVM and ROI for Site 1 (D’Or Institute for Research and Education, Rio de Janeiro), and the bottom panels show results for both SVM and ROI for Site 2 (Monash Biomedical Imaging, Monash University, Melbourne). Individual brain pictures are from t-contrasts for eight participants are set using uncorrected p < 0.05, k = 5, and masked by SVM mask for visualization purposes.


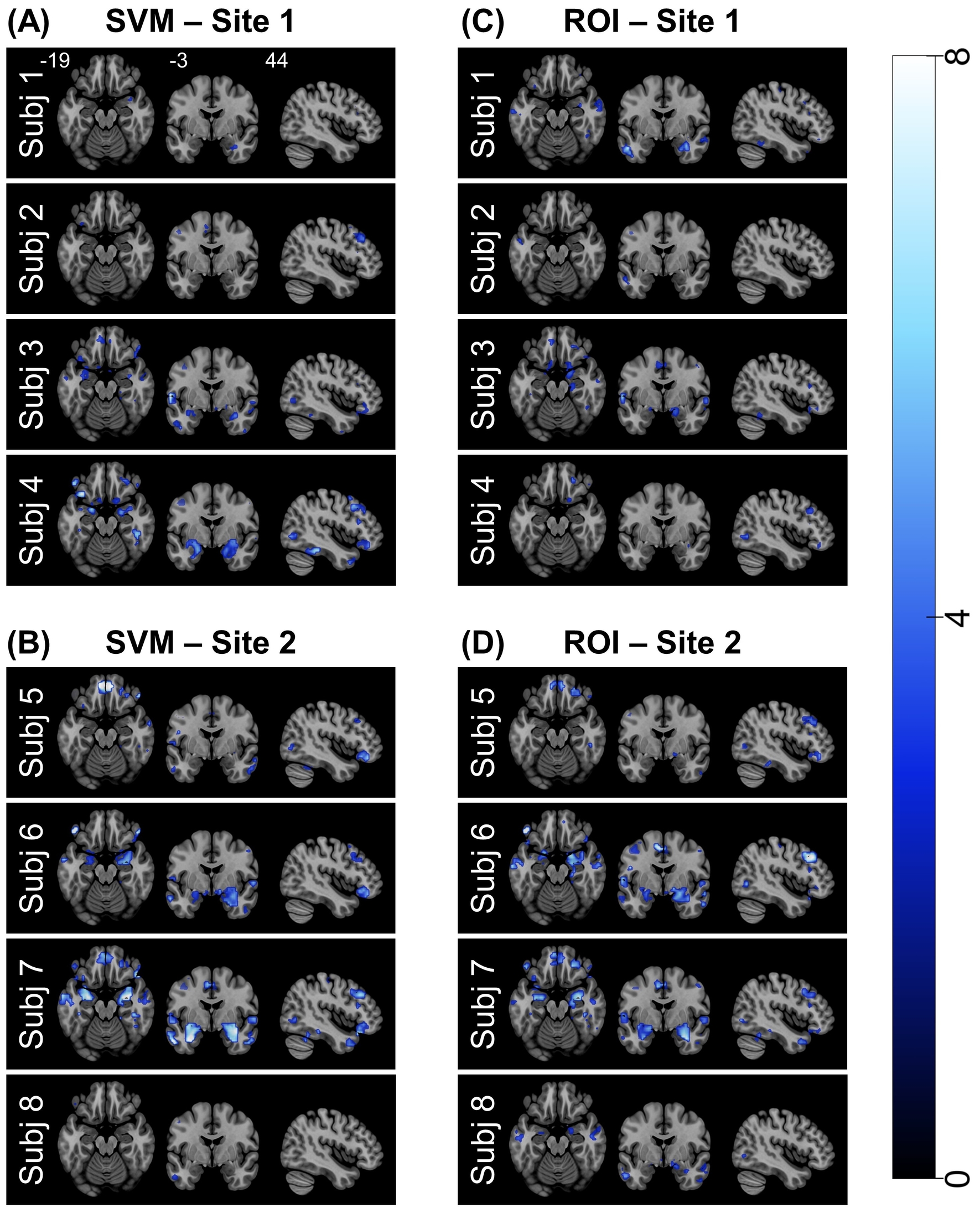


**Supplementary Figure 5.** Individual patterns of brain activity during neurofeedback Anguish > Tenderness trials using two distinct neurofeedback methods including SVM = support vector machine (left panels) and ROI = region of interest (right panels). The top panels show results for both SVM and ROI for Site 1 (D’Or Institute for Research and Education, Rio de Janeiro), and the bottom panels show results for both SVM and ROI for Site 2 (Monash Biomedical Imaging, Monash University, Melbourne). Individual brain pictures are from t-contrasts for eight participants are set using uncorrected p < 0.05, k = 5, and masked by SVM mask for visualization purposes.

## Supplementary Tables

**Supplementary Table 1.** Participants’ visual analogue scale (VAS) rating of subjective experiences across training and neurofeedback runs.

| VAS ratings | Trial type | | Training run | Neurofeedback runs 1^st^ 2^nd^ 3^rd^ | | | F, df, p neurofeedback run | site |
| --- | --- | --- | --- | --- | --- | --- | --- | --- |
| Emotion intensity | | tenderness | 2.88 (1.01) | 2.81 (1.17) | 3.50 (0.53) | 2.75 (1.13) | F=1.95, df=1, p=.19 | **F=33.14, df=1,p<.001* *(Site 2 > Site 1)*** |
|  |  | anguish | 2.88 (1.02) | 2.94 (1.06) | 2.81 (1.38) | 2.81 (1.28) | F=0.23, df=1, p=.64 | **F=11.21, df=1, p<.01* *(Site 2 > Site 1)*** |
|  |  | neutral | 2.67 (1.40) | 2.60 (1.12) | 2.73 (1.28) | 2.80 (1.08) | F=0.59, df=3, p=.62 | **F=17.99, df=1, p<.01* *(Site 2 > Site 1)*** |
| How useful emotion strategies | | tenderness | 2.93 (1.43) | 2.69 (1.44) | 2.29 (1.58) | 2.75 (1.52) | F=0.50, df=1,p=.49) | **F=69.75, df=1, p<.001* *(Site 2 > Site 1)*** |
|  |  | anguish | 2.69 (1.20) | 2.75 (1.34) | 2.81 (1.60) | 2.69 (1.54) | F=.01, df=1, p=.94) | **F=41.09, df=1, p<.001**(Site 2 > Site 1)*** |
|  |  | neutral | 2.40 (1.40) | 2.67 (1.40) | 2.60 (1.40) | 2.80 (1.42) | F=1.66, df=1, p=.22 | **F=71.48, df=1, <.001* *(Site 2 > Site 1)*** |
| Virtual environment aids emotion | | tenderness | 2.62 (1.41) | 2.69 (1.01) | 2.75 (0.93) | 2.50 (1.03) | F=0.97, df=1, p=.76 | F=4.54, df=1, p=.05 |
|  |  | anguish | 2.75 (1.24) | 3.13 (1.02) | 3.00 (0.89) | 2.88 (1.15) | F=0.07, df=1, p=.80 | F=4.10, df=1, p=.06 |
|  |  | neutral | 2.73 (1.33) | 2.93 (1.38) | 3.07 (1.22) | 3.00 (1.20) | F=1.37, df=1, p=.27 | **F=41.61, df=1, p<.001* *(Site 1 > Site 2) (sitSite 2)*Melbourne)** |
| Detect color change in virtual environment | | tenderness | n/a | 3.25 (1.65) | 3.19 (1.68) | 3.38 (1.63) | F=0.20, df=1, p=.66 | **F=19.99, df=1, p<.01* *(Site 1 > Site 2)*** |
|  |  | anguish | n/a | 3.25 (1.69) | 2.94 (1.73) | 3.25 (1.73) | F<0.01, df=1, p=.10 | **F=2.60, df=1, p<.001 *(Site 1 > Site 2)*** |
| Tiredness | | | 3.31 (1.40) | 3.19 (1.22) | 3.13 (1.09) | 2.75 (0.68) | F=2.33, df=1, p=.15 | **F=6.94, df=1, p<.05* *(Site 1 > Site 2)*** |
| Focus | | | 2.94 (1.24) | 3.06 (1.39) | 2.88 (1.31) | 3.00 (1.21) | F=0.39, df=3, p=.77 | **F=51.86, df=1, p<.001* *(Site 2 > Site 1)*** |

VAS = visual analogue scale; Site 1 = D’Or Institute for Research and Education, Rio de Janeiro; Site 2 = Monash Biomedical Imaging, Monash University, Melbourne. Ratings include participants’ experienced emotion intensity during Tenderness, Anguish and Neutral trials (from 1 = very mild to 5 = very intense), (ii) how useful they found to use the emotion regulation strategies (from 1 = very little to 5 = very useful), (iii) how easy it was to use the virtual environment during Neutral, Anguish, tenderness and Neutral trials (from 1 = extremely difficult and 5 = extremely easy), (iv) how easy they found to change the color of the virtual environment NFB interface (from 1 = extremely difficult and 5 = extremely easy), (v) fatigue (from 1= not at all and 5 = extremely) and (vi) focus (from 1= not at all and 5 = extremely).

**Supplementary Table 2.** Summary of ratings post-assessment, of music excerpts played during Tenderness and Anguish conditions.

| Emotions to rate | Musical excerpts # | | | | | | | | T (df), p |
| --- | --- | --- | --- | --- | --- | --- | --- | --- | --- |
|  | Tenderness | | | | Anguish | | | |  |
|  | i | ii | iii | iv | i | ii | iii | iv | **tenderness vs anguish** |
| tenderness | 4.00 (0.76) | 3.63 (0.74) | 3.63 (0.92) | 4.25 (0.46) | 1.00 (0.00) | 1.00 (0.00) | 1.00 (0.00) | 1.00 (0.00) | **12.69 (14), p < .001** |
| \| enchantment / admiration \| \| --- \| | 3.13 (0.99) | 3.38 (0.74) | 3.50 (0.93) | 3.88 (1.25) | 1.00 (0.00) | 1.00 (0.00) | 1.00 (0.00) | 1.00 (0.00) | **7.22 (12), p < .001** |
| transcendence | 3.50 (1.07) | 3.50 (0.76) | 3.13 (0.99) | 3.25 (1.16) | 1.25 (0.46) | 1.00 (0.00) | 1.13 (0.35) | 1.13 (0.35) | **7.83 (14), p < .001** |
| strength | 2.88 (1.13) | 2.38 (1.19) | 2.38 (0.74) | 2.88 (1.13) | 1.63 (0.74) | 1.63 (0.74) | 1.63 (0.74) | 1.50 (1.07) | **2.29 (14), p < .05** |
| serenity/peace | 3.88 (1.13) | 3.50 (0.76) | 4.00 (1.07) | 3.88 (0.83) | 1.00 (0.00) | 1.13 (0.35) | 1.00 (0.00) | 1.00 (0.00) | **10.07 (14), p < .001** |
| joy | 2.50 (0.93) | 2.25 (1.04) | 3.13 (1.13) | 3.50 (0.53) | 1.00 (0.00) | 1.00 (0.00) | 1.00 (0.00) | 1.00 (0.00) | **7.26 (14), p < .001** |
| anguish | 1.00 (0.00) | 1.00 (0.00) | 1.00 (0.00) | 1.00 (0.00) | 3.63 (1.06) | 4.13 (0.83) | 3.75 (1.16) | 3.63 (1.19) | **-8.46 (14), p < .001** |
| nostalgic | 3.50 (1.07) | 3.63 (1.06) | 3.88 (0.99) | 3.88 (1.25) | 1.13 (0.35) | 1.00 (0.00) | 1.00 (0.00) | 1.13 (0.35) | **7.71 (14), p < .001** |
| sadness | 1.75 (0.89) | 1.25 (0.46) | 1.75 (0.89) | 1.57 (0.53) | 2.25 (1.04) | 2.63 (1.06) | 2.38 (0.92) | 2.50 (0.93) | -2.04 (13), .06 |
| tension | 1.00 (0.00) | 1.00 (0.00) | 1.00 (0.00) | 1.00 (0.00) | 3.63 (1.19) | 3.88 (0.99) | 3.75 (1.04) | 3.63 (0.92) | **-7.95 (14), p < .001** |

VAS = visual analogue scale; Site 1 = D’Or Institute for Research and Education, Rio de Janeiro; Site 2 = Monash Biomedical Imaging, Monash University, Melbourne. Ratings include participants’ experienced emotion intensity during Tenderness, Anguish and Neutral trials (from 1 = very mild to 5 = very intense), (ii) how useful they found to use the emotion regulation strategies (from 1 = very little to 5 = very useful), (iii) how easy it was to use the virtual environment during Neutral, Anguish, tenderness and Neutral trials (from 1 = extremely difficult and 5 = extremely easy), (iv) how easy they found to change the color of the virtual environment NFB interface (from 1 = extremely difficult and 5 = extremely easy), (v) fatigue (from 1= not at all and 5 = extremely) and (vi) focus (from 1= not at all and 5 = extremely). Tenderness excerpts were selected from four music tracks: (i) Violin Concerto No1 Adagio by Bruch, Interp. Jascha Heifetz; (ii) Concerto Per Violino No 4 Rondo by Music Therapy; (iii) Largo from Concerto for Oboe, Strings, Basso Continuo in D by Leonard Bernstein; (iv) Las 4 Estaciones Portenas IV Invierno Porteno by Bragato, Munich Piano Trio. Matching anguish excerpts were created by editing the tenderness excerpts (one tone above and a tritone below (Koelsch et al., 2006)).

**Supplementary Table 3.** Overview of local maxima for brain activity during Tenderness versus Anguish neurofeedback conditions, by experimental site.

| **Brain area** | **Local maxima** | | **MNI Coordinates** |  |  |
| --- | --- | --- | --- | --- | --- |
|  | **Extent** | **t-value** | **x** | **y** | **z** |
| **Site 1 (IDOR, Rio de Janeiro)** |  |  |  |  |  |
| Frontal medial cortex | 241 | 10,034 | -6 | 47 | -13 |
| Precuneous | 245 | 9,394 | 3 | -58 | 32 |
| Fusiform cortex, temporal, right* | 14 | 8,310 | 42 | -7 | -25 |
| Parahippocampal gyrus, left* | 18 | 7,675 | -30 | -7 | -28 |
| Frontal pole | 12 | 6,970 | -24 | 62 | -13 |
| Temporal pole, right* | 19 | 6,896 | 33 | 5 | -34 |
| Pallidum, right | 12 | 6,624 | 15 | -1 | -4 |
| Middle temporal gyrus, posterior | 9 | 6,595 | -63 | -16 | -13 |
| Lingual gyrus, right* | 11 | 6,381 | 30 | -46 | -1 |
| Hippocampus, right | 12 | 6,288 | 18 | -43 | 11 |
| Inferior temporal gyrus, left* | 5 | 5,934 | -45 | -13 | -28 |
| Middle temporal gyrus, anterior | 11 | 5,718 | 57 | 2 | -31 |
| Middle frontal gyrus | 12 | 5,619 | -33 | 20 | 44 |
| Thalamus, right* | 8 | 5,422 | 9 | -28 | 17 |
| Intracalcarine cortex | 13 | 5,327 | 12 | -79 | 5 |
| **Site 2 (Monash University, Melbourne)** |  |  |  |  |  |
| Occipital pole | 55 | 16,113 | 21 | -100 | 14 |
|  | 122 | 13,974 | -6 | -103 | 14 |
|  |  | 12,485 | -15 | -103 | -7 |
| Frontal pole | 168 | 13,667 | -9 | 62 | -19 |
|  | 5 | 8,378 | 39 | 59 | -13 |
| Occipital pole | 21 | 8,215 | 15 | -103 | -7 |
| Occipital cortex, lateral inferior | 5 | 7,618 | -60 | -64 | -4 |
| Angular gyrus | 5 | 7,492 | 66 | -46 | 20 |
| Frontal pole | 7 | 7,453 | -24 | 41 | 50 |
| Postcentral gyrus | 7 | 6,922 | 54 | -16 | 59 |
| Frontal pole | 8 | 6,474 | 21 | 65 | -7 |
| Temporal pole | 11 | 6,396 | 33 | 20 | -34 |
| Middle frontal gyrus | 29 | 6,376 | 42 | 14 | 59 |
| Occipital cortex, lateral superior | 5 | 5,969 | -39 | -82 | 35 |
| Precentral gyrus | 5 | 5,870 | -48 | -7 | 59 |
| Orbitofrontal cortex | 7 | 5,586 | 18 | 11 | -22 |

Table shows all local maxima separated by > 20 mm, surviving threshold of p < 0.05 (whole brain FWE-corrected), t > 4.7160, df = 18548, minimum extent = 5. x, y, and z =Montreal Neurological Institute coordinate in the left-right, anterior-posterior, and inferior-superior dimensions, respectively. Regions were automatically labeled using the HarvardOxford-maxprob-thr0 atlas and regions with an * were the nearest location of activations using the same atlas.

**Supplementary Table 4.** Overview of local maxima for brain activity during Tenderness versus Anguish neurofeedback conditions, by neurofeedback method.

| **Brain area** | **Local maxima** | | **MNI Coordinates** | |  |
| --- | --- | --- | --- | --- | --- |
|  | **Extent** | **t-value** | **x** | **y** | **z** |
| **ROI** |  |  |  |  |  |
| Occipital pole | 100 | 11,519 | -6 | -103 | 14 |
|  |  | 8,952 | -15 | -103 | -7 |
|  | 41 | 11,491 | 21 | -100 | 14 |
| Frontal pole | 31 | 6,975 | -15 | 68 | -4 |
| Occipital pole | 25 | 6,748 | 30 | -97 | -7 |
| Frontal pole | 10 | 6,303 | -24 | 44 | 47 |
| Middle frontal gyrus | 8 | 5,563 | 45 | 14 | 56 |
| Frontal pole | 6 | 7,570 | 39 | 59 | -13 |
| Frontal pole | 5 | 5,249 | 3 | 62 | -10 |
| **SVM** |  |  |  |  |  |
| Frontal pole | 200 | 11,260 | 3 | 62 | -7 |
| Superior temporal gyrus, posterior | 16 | 10,070 | -69 | -28 | 5 |
| Intracalcarine cortex | 23 | 7,665 | 12 | -79 | 2 |
| Occipital cortex, lateral superior | 15 | 7,613 | -36 | -79 | 41 |
| Occipital pole | 31 | 7,185 | -21 | -103 | -1 |
| Temporal pole | 29 | 6,663 | 30 | 20 | -34 |
| Frontal pole | 9 | 6,233 | 24 | 65 | -7 |
| Precuneus | 39 | 6,126 | 9 | -55 | 26 |
| Occipital pole | 6 | 5,969 | 24 | -97 | 14 |
| Caudate. left | 7 | 5,790 | -9 | 23 | 2 |

Table shows all local maxima separated by > 20 mm, surviving threshold of p < 0.05 (whole brain FWE-corrected), t > 4.7160, df = 18548, minimum extent = 5.. x, y, and z =Montreal Neurological Institute coordinate in the left-right, anterior-posterior, and inferior-superior dimensions, respectively. Regions were automatically labeled using the HarvardOxford-maxprob-thr0 atlas and regions with an * were the nearest location of activations using the same atlas.

**Supplementary Table 5.** Overview of local maxima for brain activity during Anguish versus Tenderness neurofeedback conditions, by experimental site.

| **Brain area** | **Local maxima** | | **MNI coordinates** | | |
| --- | --- | --- | --- | --- | --- |
|  | **Extent** | **t-value** | **x** | **y** | **z** |
| **Site 1 ( Rio de Janeiro)** |  |  |  |  |  |
| Supramarginal gyrus, posterior | 1293 | 10,134 | -60 | -43 | 20 |
| Precentral gyrus |  | 9,722 | -48 | 5 | 14 |
|  |  | 8,505 | -42 | -1 | 35 |
| Precentral gyrus | 735 | 9,941 | 54 | 8 | 20 |
| Frontal pole |  | 8,179 | 42 | 44 | 23 |
| Frontal operculum |  | 7,643 | 36 | 26 | 2 |
| Postcentral gyrus | 359 | 8,343 | 57 | -19 | 44 |
| Supramarginal gyrus, posterior |  | 7,158 | 45 | -40 | 47 |
|  |  | 6,826 | 63 | -37 | 14 |
| Lingual gyrus | 275 | 10,079 | -18 | -61 | -10 |
| Fusiform cortex, temporal occipital |  | 7,286 | -30 | -46 | -22 |
| Inferior temporal gyrus |  | 5,034 | -48 | -58 | -7 |
| Occipital cortex, lateral superior | 168 | 8,717 | 30 | -76 | 41 |
|  |  | 7,106 | 9 | -73 | 62 |
| Lingual gyrus | 129 | 6,916 | 24 | -64 | -7 |
| Insula | 50 | 8,674 | 36 | -4 | 5 |
| Amygdala, right | 50 | 5,238 | 33 | -7 | -7 |
| Superior frontal gyrus | 43 | 7,033 | 3 | 23 | 53 |
| Occipital cortex, lateral superior | 37 | 6,488 | -27 | -70 | 29 |
| Superior parietal lobule | 36 | 6,093 | -36 | -46 | 47 |
| Occipital cortex, lateral* | 32 | 6,220 | 15 | -61 | 53 |
| Supplementary motor area** | 30 | 7,030 | 3 | 5 | 53 |
| Occipital cortex, lateral superior | 30 | 6,062 | -15 | -76 | 56 |
| Middle frontal gyrus | 28 | 6,524 | -48 | 29 | 23 |
| Occipital cortex, lateral superior | 25 | 6,319 | 48 | -70 | 17 |
| Putamen, left | 21 | 6,735 | -30 | -4 | 2 |
| Superior frontal gyrus | 21 | 5,591 | 24 | -4 | 56 |
| Middle temporal gyrus, posterior | 20 | 7,697 | 48 | -19 | -10 |
| Middle temporal gyrus, left* | 20 | 7,514 | -45 | -4 | -28 |
| Orbitofrontal cortex | 18 | 6,659 | 24 | 23 | -19 |
| Occipital cortex, left* | 18 | 6,015 | -33 | -82 | 11 |
| Frontal pole | 16 | 6,227 | -24 | 44 | 20 |
| Occipital cortex, lateral inferior | 16 | 5,957 | 51 | -61 | 5 |
| Inferior temporal gyrus | 14 | 6,051 | 48 | -46 | -19 |
| Insula | 13 | 6,327 | -39 | -4 | 5 |
| Superior frontal gyrus, left* | 12 | 5,878 | -21 | -10 | 50 |
| Frontal pole | 12 | 5,525 | 45 | 53 | 5 |
| Precentral gyrus | 10 | 5,644 | -36 | -4 | 53 |
| Cerebral cortex, right* | 9 | 5,908 | 9 | 5 | 29 |
| Precentral gyrus, right* | 8 | 5,325 | 42 | -10 | 38 |
| Middle temporal gyrus, right * | 7 | 5,635 | 51 | -52 | -4 |
| Precentral gyrus | 7 | 5,374 | -42 | -13 | 56 |
| Inferior temporal gyrus, right* | 7 | 5,080 | 42 | -61 | -7 |
| Occipital cortex, lateral inferior | 6 | 5,874 | 45 | -73 | -7 |
| Middle frontal gyrus, right* | 6 | 5,502 | 30 | 26 | 29 |
| Pallidum, right | 5 | 6,326 | 24 | -7 | 2 |
| Occipital cortex, lateral inferior | 5 | 5,166 | -45 | -67 | -1 |
| Inferior temporal gyrus | 5 | 5,159 | -51 | -52 | -25 |
| **Site 2 (Melbourne)** |  |  |  |  |  |
| Frontal pole | 19556 | 18,686 | -42 | 44 | -13 |
| Occiptal cortex, lateral, left* |  | 17,082 | -21 | -82 | 29 |
| Superior frontal gyrus |  | 15,849 | -6 | 26 | 62 |
| Amygdala, right |  | 8,991 | 30 | -7 | -22 |
| Middle temporal gyrus, anterior | 50 | 9,019 | 51 | 2 | -37 |
|  | 37 | 7,436 | -63 | -4 | -22 |
| Fusiform cortex, temporal, posterior | 22 | 6,470 | -42 | -25 | -25 |
| Cingulate gyrus, right* | 19 | 6,048 | 15 | -46 | 29 |
| Frontal pole | 8 | 6,851 | -6 | 56 | -16 |
| Orbitofrontal cortex | 7 | 5,586 | 18 | 11 | -22 |
| Superior temporal gyrus, anterior | 5 | 6,026 | 60 | -1 | -13 |

Table shows all local maxima separated by > 20 mm, surviving threshold of p < 0.05 (whole brain FWE-corrected), t > 4.7160, df = 18548, minimum extent = 5. x, y, and z =Montreal Neurological Institute coordinate in the left-right, anterior-posterior, and inferior-superior dimensions, respectively. Regions were automatically labeled using the HarvardOxford-maxprob-thr0 atlas and regions with an * were the nearest location of activations using the same atlas, ** Juxtapositional lobule cortex

**Supplementary Table 6.** Overview of local maxima for brain activity during Anguish versus Tenderness neurofeedback conditions, by neurofeedback method.

| **Brain area** | **Local maxima** | | **MNI coordinates** | | |
| --- | --- | --- | --- | --- | --- |
|  | **Extent** | **t-value** | **x** | **y** | **z** |
| **ROI** |  |  |  |  |  |
| Occipital cortex, lateral superior | 8134 | 11,548 | 24 | -79 | 44 |
| Occipital cortex, lateral superior, left* |  | 11,470 | -24 | -79 | 29 |
| Supramarginal gyrus, anterior |  | 11,443 | 66 | -31 | 26 |
| Amygdala, right |  | 6,984 | 24 | -10 | -13 |
| Superior parietal lobule | 54 | 8,635 | -18 | -52 | 65 |
| Superior frontal gyrus | 105 | 8,550 | 0 | 11 | 65 |
|  |  | 6,997 | 24 | 17 | 59 |
| Precentral gyrus | 76 | 8,539 | -27 | -19 | 65 |
|  | 43 | 8,243 | -3 | -31 | 68 |
|  | 43 | 7,408 | 24 | -31 | 68 |
| Frontal pole | 48 | 7,602 | 9 | 71 | 14 |
| Subcallosal cortex | 39 | 7,294 | -9 | 26 | -22 |
| Cingulate gyrus, right* | 242 | 6,704 | 6 | 14 | 23 |
| Superior frontal gyrus |  | 6,498 | 0 | 47 | 41 |
|  |  | 6,343 | 3 | 23 | 50 |
| Amygdala, right* | 9 | 6,647 | 30 | -1 | -28 |
| Cingulate gyrus, right* | 19 | 6,597 | 3 | -40 | 8 |
| Cerebral córtex, left* | 22 | 6,566 | -45 | -4 | -28 |
| Frontal pole | 11 | 6,279 | -24 | 65 | 2 |
| Intracalcarine cortex | 16 | 6,199 | -9 | -82 | 11 |
| Superior temporal gyrus, posterior | 13 | 6,101 | -48 | -13 | -13 |
| Frontal pole | 17 | 6,047 | 27 | 68 | -1 |
| Supplementary motor córtex** | 53 | 6,018 | 3 | 2 | 50 |
| Cingulate gyrus, posterior | 26 | 5,997 | 12 | -34 | 41 |
| Putamen, left | 107 | 5,943 | -30 | -4 | -1 |
| Cerebral cortex, left* |  | 5,672 | -27 | 20 | 2 |
| Pallidum, left | 70 | 5,941 | -21 | -1 | -1 |
| Thalamus, left | 10 | 5,917 | -6 | -13 | -4 |
| Precuneous, right* | 22 | 5,905 | 24 | -52 | 11 |
| Occipital cortex, lateral inferior | 12 | 5,878 | -39 | -85 | -22 |
| Middle frontal gyrus | 10 | 5,865 | -30 | 29 | 41 |
| Occipital cortex, lateral inferior | 10 | 5,808 | 45 | -70 | -25 |
| Cerebral cortex, left* | 7 | 5,682 | -33 | -31 | 38 |
| Putamen, left* | 7 | 5,679 | -33 | -22 | -4 |
| Precentral gyrus, right* | 6 | 5,588 | 18 | -22 | 59 |
| Frontal pole | 9 | 5,570 | -9 | 65 | 11 |
| Thalamus, left | 6 | 5,508 | -6 | -7 | 11 |
| Middle temporal gyrus, right* | 16 | 5,328 | 45 | -19 | -10 |
| Cingulate gyrus, left* | 18 | 5,325 | -3 | -16 | 26 |
| Superior parietal lobule | 6 | 5,324 | 30 | -55 | 62 |
| Thalamus, right* | 5 | 5,202 | 15 | -1 | 11 |
| Fusiform gyrus, occipital | 10 | 5,201 | -24 | -85 | -22 |
| **SVM** |  |  |  |  |  |
| Lingual gyrus | 4040 | 12,408 | -9 | -76 | -13 |
| Occipital cortex, left* |  | 11,913 | -21 | -82 | 29 |
| Fusiform gyrus, occipital |  | 10,669 | -33 | -73 | -22 |
| Frontal pole | 5938 | 12,261 | -36 | 41 | -13 |
| Precentral gyrus |  | 12,037 | 57 | -1 | 35 |
| Frontal pole |  | 10,895 | 42 | 41 | -13 |
| Amygdala, right |  | 6,433 | 33 | -4 | -7 |
| Supramarginal gyrus, right* | 60 | 7,435 | 54 | -34 | 38 |
| Thalamus, right* | 60 | 7,093 | 12 | -13 | -7 |
| Brain-stem | 60 | 5,471 | 0 | -28 | -1 |
| Superior temporal gyrus, posterior | 11 | 6,551 | 69 | -13 | -1 |
| Frontal pole | 10 | 6,528 | 9 | 47 | -22 |
| Parietal operculum | 29 | 6,210 | -36 | -31 | 20 |
| Orbitofrontal cortex | 6 | 6,120 | -33 | 20 | -19 |
| Frontal pole | 8 | 6,114 | 39 | 62 | 8 |
| Supramarginal gyrus, posterior | 11 | 6,088 | 57 | -43 | 26 |
| Superior parietal lobule | 10 | 5,938 | 39 | -43 | 68 |
| Cingulate gyrus, posterior | 22 | 5,678 | -3 | -19 | 29 |
| Brain-stem | 9 | 5,333 | -9 | -25 | -22 |
| Fusiform cortex, temporal posterior | 5 | 5,328 | 33 | -25 | -25 |
| Middle temporal gyrus, posterior | 16 | 5,313 | -54 | -22 | -7 |
| Superior parietal lobule, right* | 5 | 5,064 | 27 | -40 | 41 |
| Putamen, right* | 7 | 5,030 | 27 | -25 | 11 |

Table shows all local maxima separated by > 20 mm, surviving threshold of p < 0.05 (whole brain FWE-corrected), t > 4.7160, df = 18548, minimum extent = 5. x, y, and z =Montreal Neurological Institute coordinate in the left-right, anterior-posterior, and inferior-superior dimensions, respectively. Regions were automatically labeled using the HarvardOxford-maxprob-thr0 atlas and regions with an * were the nearest location of activations using the same atlas; ** Juxtapositional lobule cortex.
